# Warming effects on grassland soil microbial communities are amplified in cool months

**DOI:** 10.1093/ismejo/wrae088

**Published:** 2024-05-15

**Authors:** Jiesi Lei, Yuanlong Su, Siyang Jian, Xue Guo, Mengting Yuan, Colin T Bates, Zhou Jason Shi, Jiabao Li, Yifan Su, Daliang Ning, Liyou Wu, Jizhong Zhou, Yunfeng Yang

**Affiliations:** State Key Joint Laboratory of Environment Simulation and Pollution Control, School of Environment, Tsinghua University, Beijing 100084, China; State Key Joint Laboratory of Environment Simulation and Pollution Control, School of Environment, Tsinghua University, Beijing 100084, China; Institute for Environmental Genomics and Department of Microbiology and Plant Biology, University of Oklahoma, Norman, OK 73019, United States; State Key Joint Laboratory of Environment Simulation and Pollution Control, School of Environment, Tsinghua University, Beijing 100084, China; State Key Laboratory of Urban and Regional Ecology, Research Center for Eco-Environmental Sciences, Chinese Academy of Sciences, Beijing 100085, China; Institute for Environmental Genomics and Department of Microbiology and Plant Biology, University of Oklahoma, Norman, OK 73019, United States; Department of Environmental Science, Policy, and Management, University of California, Berkeley, CA 94704, United States; Institute for Environmental Genomics and Department of Microbiology and Plant Biology, University of Oklahoma, Norman, OK 73019, United States; Institute for Environmental Genomics and Department of Microbiology and Plant Biology, University of Oklahoma, Norman, OK 73019, United States; Key Laboratory of Environmental and Applied Microbiology, Chinese Academy of Sciences and Environmental Microbiology & Key Laboratory of Sichuan Province, Chengdu Institute of Biology, Chinese Academy of Sciences, Chengdu 610213, China; State Key Joint Laboratory of Environment Simulation and Pollution Control, School of Environment, Tsinghua University, Beijing 100084, China; Institute for Environmental Genomics and Department of Microbiology and Plant Biology, University of Oklahoma, Norman, OK 73019, United States; Institute for Environmental Genomics and Department of Microbiology and Plant Biology, University of Oklahoma, Norman, OK 73019, United States; Institute for Environmental Genomics and Department of Microbiology and Plant Biology, University of Oklahoma, Norman, OK 73019, United States; School of Civil Engineering and Environmental Sciences, University of Oklahoma, Norman, OK 73019, United States; Earth and Environmental Sciences Area, Lawrence Berkeley National Laboratory, Berkeley, CA 94720, United States; State Key Joint Laboratory of Environment Simulation and Pollution Control, School of Environment, Tsinghua University, Beijing 100084, China; Institute of Environment and Ecology, Tsinghua Shenzhen International Graduate School, Tsinghua University, Shenzhen 518055, China

**Keywords:** soil respiration, microbial functional traits, seasonal succession, global warming, carbon decomposition

## Abstract

Global warming modulates soil respiration (*R*_S_) via microbial decomposition, which is seasonally dependent. Yet, the magnitude and direction of this modulation remain unclear, partly owing to the lack of knowledge on how microorganisms respond to seasonal changes. Here, we investigated the temporal dynamics of soil microbial communities over 12 consecutive months under experimental warming in a tallgrass prairie ecosystem. The interplay between warming and time altered (*P* < 0.05) the taxonomic and functional compositions of microbial communities. During the cool months (January to February and October to December), warming induced a soil microbiome with a higher genomic potential for carbon decomposition, community-level ribosomal RNA operon (*rrn*) copy numbers, and microbial metabolic quotients, suggesting that warming stimulated fast-growing microorganisms that enhanced carbon decomposition. Modeling analyses further showed that warming reduced the temperature sensitivity of microbial carbon use efficiency (CUE) by 28.7% when monthly average temperature was low, resulting in lower microbial CUE and higher heterotrophic respiration (*R*_h_) potentials. Structural equation modeling showed that warming modulated both *R*_h_ and *R*_S_ directly by altering soil temperature and indirectly by influencing microbial community traits, soil moisture, nitrate content, soil pH, and gross primary productivity. The modulation of *R*_h_ by warming was more pronounced in cooler months compared to warmer ones. Together, our findings reveal distinct warming-induced effects on microbial functional traits in cool months, challenging the norm of soil sampling only in the peak growing season, and advancing our mechanistic understanding of the seasonal pattern of *R*_S_ and *R*_h_ sensitivity to warming.

## Introduction

The rising global surface temperature is one of the most remarkable climate changes faced by the terrestrial ecosystem [1], profoundly affecting terrestrial carbon (C) dynamics and ecosystem-atmosphere C exchanges [2]. Rising temperature is expected to accelerate soil C loss or soil respiration (*R*_S_) [3] by enhancing the mineralization of soil organic C through heterotrophic respiration (*R*_h_, mainly contributed by soil microorganisms and animals) or autotrophic respiration (*R*_a_, mainly contributed by plant roots and rhizosphere), which are two primary components of *R*_S_ [4]. However, the increase in *R*_S_ can be offset towards ambient values due to various factors, including shifts in plant community composition, reduced photosynthetic rates in ecosystems not limited by temperature [5], the thermal adaptation of microorganisms, depletion of soil organic C, and decrease in soil moisture [6]. Therefore, it is imperative to determine the dynamics of *R*_S_ or *R*_h_ and their underlying mechanisms in a warming climate.

Soil microorganisms are key drivers of the decomposition of organic matter, thereby influencing soil C stocks and fluxes [4]. In grassland ecosystems, which are significant reservoirs of soil C [2], changes in microbial community composition and function due to warming can profoundly impact ecosystem services such as soil fertility, plant productivity, and C sequestration [7]. Given the pronounced sensitivity of grassland ecosystems to climate change [8], our study zeroes in on warming—a critical component of climate change—to understand its specific effects on microbial-mediated processes.

Although there is ample literature on the long-term responses of soil microbial communities to warming, only a handful has considered the effect of seasonal variation [9, 10]. However, it is crucial to take seasonal variation into account because biotic (e.g. plants and animals) and abiotic (e.g. moisture and nutrient availability) components of natural ecosystems usually exhibit strong phenological patterns and seasonality [11–14]. As temperature is one of the most important determinants of *R*_h_ [15], we posit that warming disproportionately influences *R*_h_ due to a combination of factors that vary throughout the year such as microbial community composition, functional traits, soil moisture levels, and substrate availability. During cooler months, we hypothesize that lower baseline temperatures and the presence of unutilized C substrates from the preceding growing season favor fast-growing, *r*-strategist microorganisms with high genomic potential for C decomposition. These organisms could exhibit enhanced metabolic activity in response to warming, leading to increased soil C loss through *R*_h_.

## Materials and methods

### Site description and soil sampling

The experimental study was carried out at the KAEFS located in McClain County, Oklahoma, USA (ca. 34^o^59′N, 97^o^3′W; Fig. S1). The experimental site is a mixed-grass prairie dominated by C_3_ forbs (*Ambrosia trifida*, *Solanum carolinense*, and *Euphorbia dentata*), C_3_ grass (*Bromus japonicus Thunb.*), and C_4_ grass (*Tridens flavus*, *Sporobolus compositus*, and *Sorghum halapense*). As described previously [16, 17], the mean annual temperature of this area from 1948 to 2012 was 16.3°C, with mean monthly air temperature ranging from 3.5°C in January to 28.1°C in July. The mean annual precipitation was 895 mm, with monthly precipitation ranging from 33 mm in January to 126 mm in May (Oklahoma Climatological Survey, Norman, OK, USA). Annual peak plant biomass was attained from late April to early May when C3 plants dominated plant communities and late August to early September when C4 plants dominated plant communities. The soil type was the Port–Pulaski–Keokuk complex, with a neutral pH, a high available water-holding capacity (37%), and a deep (~70 cm), moderately penetrable root zone. The soil texture is loam with 51% sand, 35% silt, and 13% clay.

The experiment to simulate global warming was initiated in July 2009. There were four biological replicate blocks. Each block is divided into two 2.5 × 1.75 m plots, with one plot designated as the ambient control and the other as the warming treatment (an average of +3.0°C throughout the year) in a paired design. Two infrared heaters (165 × 9 × 15 cm; Kalglo Electronics, Bethlehem, PA, USA) were installed at an approximate height of 1.5 m above each warmed plot to heat the soil evenly. Two dummy heaters, identical in dimensions, were suspended above the control plots to mimic the shading effects.

One surface soil (0–15 cm) sample core was collected on a monthly basis for each of the eight warmed and control plots in 2012 (the third year of experimental manipulation). In total, 96 monthly samples were collected. A portion of the soil was immediately frozen at −80°C for molecular analyses, and the rest of the soil was oven-dried for physicochemical analyses.

### Field survey and soil physical–chemical analyses

Soil temperature was measured automatically every 15 min with thermocouples (T-type; Campbell Science Inst., Logan, UT, USA) installed at a depth of 7.5 cm at the center of each main plot to reflect the mean topsoil temperature. Volumetric soil water content (%V) in the top 15 cm soil was measured automatically every 30 min with time domain reflectometry (TDR) meters (ESI Environmental Sensors Inc., Sidney, BC, Canada) installed in each plot. Monthly soil temperature and moisture were calculated from corresponding time-series measurements. Ecosystem C exchanges were measured on a monthly basis between 10:00 and 15:00 local time on sunny days using an LI-6400 (LI-COR Biosciences, Lincoln, NE, USA) Portable Photosynthesis System with a transparent chamber (0.5 m × 0.5 m × 0.7 m, with fans circulating the air inside), aligning with the methodologies of previous studies [18–21]. The chamber was placed and sealed on a metal frame in the plot, and covered all the vegetation inside the frame. Net ecosystem C exchange (NEE) was calculated using C fluxes measured with a chamber exposed to sunlight, whereas ecosystem respiration (*R*_e_) was retrieved from the C fluxes measured when the chamber was kept in the dark with a light-proof cover. Gross primary production (GPP) was calculated as the difference between NEE and *R*_e_. *R*_S_ and *R*_h_ were measured on a monthly basis between 10:00 and 15:00 local time using an LI-8100A soil flux system attached to a soil CO_2_ flux chamber (LI-COR Biosciences, Lincoln, NE, USA) [16]. Measurements were taken above a PVC collar (80 cm^2^ in area and 5 cm in depth) and a PVC tube (80 cm^2^ in area and 70 cm in depth) permanently fixed at the center of each plot. Old plant roots inside the PVC tube were cut to prevent root growth. The aboveground parts of living plants were removed from the PVC tubes and collars before each measurement. CO_2_ flux measured above the PVC tube represented *R*_h_ from microbial metabolism due to root exclusion, and that measured above the PVC collar included both *R*_h_ and *R*_a_ from soil microorganisms and plant roots, representing soil *R*_S_. *R*_a_ was calculated as the difference between *R*_S_ and *R*_h_.

Oven-dried soil samples were ground and analyzed for total C, total N, ammonia NH_4_^+^, and nitrate NO_3_^−^ contents by the Soil, Water, and Forage Analytical Laboratory (SWFAL) at Oklahoma State University, Stillwater, OK, USA. A dry combustion C and nitrogen (N) analyzer (LECO, St. Joesph, MI, USA) was used to quantify total C and total N. Soil NH_4_^+^ and NO_3_^−^ contents were measured by a Lachat 8000 flow-injection analyzer (Lachat, Milwaukee, WI, USA).

### DNA extraction and Illumina sequencing

Soil DNA was extracted and purified according to the previous protocols [22]. DNA concentrations were quantified with PicoGreen using a FLUOstar OPTIMA fluorescence plate reader (BMG LabTech, Jena, Germany), and DNA purity was determined by the ratios of O.D. 260/280 nm and O.D. 260/230 nm using a NanoDrop ND-1000 Spectrophotometer (NanoDrop Technologies Inc., Wilmington, DE, USA). Because DNA yield showed strong, positive correlations with microbial C in soils [23–25] and can reliably indicate microbial biomass across a wide range of soil types and ecosystems [26, 27], we used DNA concentration as a proxy of microbial biomass in this study.

The V4 hypervariable region of bacterial 16S rRNA gene was amplified by PCR, using primers 515F (5′-GTG CCA GCM GCC GCG GTA A-3′) and 806R (5′-GGA CTA CHV GGG TWT CTA AT-3′) combined with adapter sequences and barcode sequences [28]. A total of 100 ng of amplicons from each sample were pooled and purified with QIAquick Gel Extraction Kit (Qiagen Inc., Venlo, The Netherlands). After purification, the amplicons were quantified in triplicate through PicoGreen, and used for sequencing library preparation following the standard Illumina protocol. The amplicon sequencing library was then sequenced on an MiSeq platform (Illumina) in 2 × 250 bp pair-end format.

Raw reads were processed using usearch v11 [29]. After demultiplexing, forward and reverse sequences were merged using -mergepairs and primer-trimmed using -search_pcr2. The sequences were then quality filtered using -fastq_filter -fastq_maxee 1.0. The sequences were dereplicated using -fastx_uniques with -sizeout -relabel Uniq. Exact amplicon sequence variants (ASVs) were generated using the UNOISE algorithm (i.e. -unoise3). ASV tables were created by mapping the raw sequence reads to the ASVs using -otutab with the -zotus and -strand options. The representative sequence of each ASV was assigned to a taxonomic lineage using Naive Bayes classifier against the SILVA ribosomal RNA (rRNA) database (release 138).

### Estimation of the rRNA operon copy number

The rRNA operon copy number for bacterial ASV was estimated through the rrnDB (Ribosomal RNA Operon Copy Number Database) version 5.7 [30] based on their closest relatives with known *rrn* copy numbers, as described previously [31]. Specifically, each ASV was matched with the database from the lowest confident (> 50%) taxonomic rank. For ASVs with available child taxon matches, the mean *rrn* copy number of the child taxa was used, otherwise, higher rank matches were searched and the mean *rrn* copy number of the parent taxa for that ASV was assigned. We calculated the community aggregated (weighted mean) *rrn* copy number for each sample by taking the product of the estimated operon copy number and the relative abundance for each ASV, and summing this value across all ASVs in a sample. Namely,


(1) \begin{equation*} \text{community}\ \text{average}\ rrn\ \text{copy}\ \text{number}=\frac{\sum_{i=1}^N{S}_i}{\sum_{i=1}^N\frac{S_i}{n_i}}, \end{equation*}

where $\text{N}$ is the number of ASVs in a sample, ${S}_i$ is the sequence abundance of ASV_i_, and ${n}_i$ is the estimated *rrn* copy number of ASV_i_.

### GeoChip hybridization and data analyses

GeoChip 5.0, a DNA microarray containing oligonucleotide probes for a large number of microbial functional genes [32], was used to assess the functional responses of microbial communities. Purified DNA extracted from each sample was labeled with Cy3 using random primers, dried, rehydrated, and hybridized with GeoChip 5.0, as described previously [33]. Subsequently, slides were rinsed and scanned with a NimbleGen MS200 microarray scanner (Roche NimbleGen, Madison, WI, USA). Probe spots with a coefficient of variance (CV) > 0.8 were discarded. Raw signals were uploaded to Microarray Data Manager (http://ieg.ou.edu/microarray) for data quality control and normalization. Samples from January to June and from July to December were hybridized in separate batches. The signal-to-noise ratio (SNR) was set to 7 for January to June samples or 3.5 for July to December samples to minimize the batch effect. Spots with minimum intensity <100 or detected in <50% of replicate samples were removed before statistical analyses. Both the universal standard and functional gene spot intensities were used to normalize the signals among arrays. Data were logarithmically transformed after quality control and normalization.

### Statistical analyses

All statistical analyses were performed using R software 4.0.2 with the packages vegan [34] and ieggr, unless otherwise specified. We assessed the local temperature variability to divide the year-round data into cool and warm seasons. Briefly, the monthly air temperature was fitted to a fifth-degree polynomial spline function, from which the first derivative was calculated representing air temperature change rates corresponding to each time point of the year. The fastest temperature rise occurred between February and March, while the fastest temperature decrease occurred between September and October. Therefore, January and February are considered the early cool season, March to September are considered the warm season, and October to December are considered the late cool season. As such, the warm season typically has monthly temperatures above ~15°C, whereas the cool seasons have monthly temperatures below 15°C.

Nonmetric multidimensional scaling (NMDS) was performed to determine microbial functional and taxonomic compositions. Microbial β-diversity was assessed by the Bray-Curtis distance metrics. Three complementary nonparametric analyses (Adonis, Mrpp, and Anosim) were used to detect the overall dissimilarity of microbial functional and taxonomic compositions under warming treatment and in different seasons. The distances of paired warmed and control plots within each block were fitted to nonlinear quadratic regression. The student’s *t*-test was used to compare community average *rrn* copy numbers between warming and control samples in each season (*n* = 8 for the early cool season, 28 for the warm season, and 12 for the late cool season). The logarithmic difference in community average *rrn* copy number was calculated with the following formula, 


\begin{equation*} \mathit{\ln}\frac{rrn\ copy\ number\ of\ A}{rrn\ copy\ number\ of\ {A}^{\prime }}, \end{equation*}

where a matching pair of samples A (warming) and A′ (control) was used.

Pearson correlations were used to analyze the relationship between warming-induced changes in the community average *rrn* copy number and soil temperature. The response ratio analysis was used to evaluate the warming effects on functional genes at sub-category and probe levels. Spearman correlation was used to test the monotonic relationship between the response ratio of C decomposition genes and warming-induced changes in community average *rrn* copy number. Because response ratios of C decomposition genes were calculated on a monthly basis, pairwise logarithmic differences in community average *rrn* copy number were also averaged across 4 blocks for each month. The analysis of variance (ANOVA) was used to analyze the effects of warming and months on soil variables, *R*_S_, ecosystem fluxes, and community average *rrn* copy number. Partial Mantel tests were used to determine correlations between the relative abundance of functional genes and *R*_S_.

Structural equation models (SEMs) were constructed with R package lavaan [35]. To ensure sufficient sample size for SEM analysis, samples from early and late cool seasons were combined and herein referred to as “the cool season” for simplicity. The initial model construction was guided by a hypothesis that warming influences ecosystem functions (*R*_S_ and *R*_h_) through alterations in environmental variables (soil temperature, soil moisture, NO_3_^−^ content, and soil pH), plant communities (GPP), and microbial communities (bacterial richness, community-level *rrn* copy number, and functional composition). The first-axis scores from NMDS were used to characterize the microbial functional composition, as it captured the primary variation in microbial functional communities. We constructed an a priori model (Fig. S2) with all reasonable pathways, then pruned nonsignificant paths unless they held biological or ecological significance, and adjusted the model based on residual correlations. The procedure was repeated until the model showed sufficient fitting, reaching *P*-values of χ^2^-test >0.05 (that is, the predicted model and observed data are not significantly different), high GFI (≥0.90), and low RMSEA (≤0.06). Upon achieving a good model fit, we interpreted the path coefficients of the model and their associated *P-*values. A path coefficient is akin to the partial correlation coefficient that describes the direction and strength of the relationship between two variables. To quantify the contributions of warming and other predictors to *R*_S_/*R*_h_ variability, we calculated the standardized total effects by summing up the direct and indirect effects, where direct effects represent immediate variable impacts, and indirect effects trace through intermediary pathways, thus providing a comprehensive view of the ecosystem’s response dynamics.

### Simulations with TECO and Microbial-ENzyme Decomposition models

The Microbial-ENzyme Decomposition (MEND) model [36] describes the SOM decomposition processes by explicitly representing relevant microbial and enzymatic physiology. Model state variables, governing equations, component fluxes, and parameters are described in Table S5. The datasets (e.g. daily GPP, soil temperature, soil mofigsture, and *R*_h_) used for data assimilation were reported in our previous study [18]. We incorporated the monthly data of microbial gene abundances associated with oxidative and hydrolytic enzymes into the model parameterization of MEND, generating a gMEND model. We selected eleven model parameters to regulate microbial processes, using default starting values [36]. These parameters include one parameter relevant to soil C input (*f*INP), three parameters relevant to enzyme production and turnover (*r_E_*, *p_EP_*, and *fp_EM_*), two parameters relevant to C flow to dissolved organic C (*f_D_* and *g_D_*), and five parameters relevant to microbial growth, maintenance, and dormancy (*V_g_*, *α*, *K_D_*, *β*, and *ψ_A2D_*).

The model parameters are determined by achieving high goodness-of-fits of model simulations against experimental observations. We implemented multiobjective calibration of model parameters [37]. Each objective evaluates the goodness-of-fit of a specific observed variable such as *R*_h_ or microbial gene abundances (Table S6). The parameter optimization is to minimize the overall objective function (J) computed as the weighted average of multiple single objectives


(2) \begin{equation*} J=\sum_{i=1}^m{w}_i\cdot{J}_i \end{equation*}

(3) \begin{equation*} \sum_{i=1}^m{w}_i=1\ \text{with}\ {w}_i\in \left[0,1\right], \end{equation*}

where *m* denotes the number of objectives, and *w*_i_ is the weighting factor for the *i*th (*i* = 1,2, …, *m*) objective (*J_i_*). In this data assimilation, *J_i_* (*i* = 1, 2, 3) is used to represent the objective function value for three different variables, namely *R*_h_, EnzCo, and EnzCh. Because we have far more *R*_h_ observations (e.g. 74 in control or warmed cases) than the other variables and *R*_h_ is the most important variable in soil C studies, we assigned a much higher weighting factor to *R*_h_ than the other two objective functions (EnzCo and EnzCh), i.e. *w*_1_ = 3/5 and *w*_2_ = *w*_3_ = 1/5.

As the overall objective function $J$ is minimized in the parameter optimization process, the individual objective function ${J}_i$ may be calculated as (1 − *R*^2^) or (1 − *r*). The coefficient of determination (*R*^2^, Eq. 4) was used to evaluate *R*_h_ because it was frequently measured, and the absolute values could be directly compared between observations and simulations. *R*^2^ quantifies the proportion of the variance in the response variables that is predictable from the independent variables. The correlation coefficient (*r*, Eq. 5) between logarithmic transformed observations and simulations was used to evaluate the goodness-of-fit for EnzCo and EnzCh because the gene abundances from metagenomics or GeoChip analysis cannot be directly compared to the enzyme concentrations or activities in the MEND model. A higher *R*^2^ (*R*^2^ ≤ 1) or *r* value (|*r*| ≤1) indicates better model performance. *n* is the number of data, ${Y}_{obs}$ are observed values, and ${Y}_{sim}$ are simulated values. ${\overline{Y}}_{obs}$ is the mean value for ${Y}_{obs}$ and ${\overline{Y}}_{sim}$ is the mean value for ${Y}_{sim}$


(4) \begin{equation*} {R}^2=1-\frac{\sum_{\text{i}=1}^{\text{n}}{\left[{\text{Y}}_{\text{sim}}\left(\text{i}\right)-{\text{Y}}_{\text{obs}}\left(\text{i}\right)\right]}^2}{\sum_{\text{i}=1}^{\text{n}}{\left[{\text{Y}}_{\text{obs}}\left(\text{i}\right)-{\overline{\text{Y}}}_{\text{obs}}\right]}^2} \end{equation*}

(5) \begin{equation*} r=\frac{\sum_{\text{i}=1}^{\text{n}}\left[{\text{Y}}_{\text{obs}}\left(\text{i}\right)-{\overline{\text{Y}}}_{\text{obs}}\right]\cdot \left[{\text{Y}}_{\text{sim}}\left(\text{i}\right)-{\overline{\text{Y}}}_{\text{sim}}\right]}{\sqrt{\sum_{\text{i}=1}^{\text{n}}{\left[{\text{Y}}_{\text{obs}}\left(\text{i}\right)-{\overline{\text{Y}}}_{\text{obs}}\right]}^2}\cdot \sqrt{\sum_{\text{i}=1}^{\text{n}}{\left[{\text{Y}}_{\text{sim}}\left(\text{i}\right)-{\overline{\text{Y}}}_{\text{sim}}\right]}^2}}. \end{equation*}

Model parameters for warming and control samples were determined with the Shuffled Complex Evolution (SCE) algorithm, and parameter uncertainty was quantified by the Critical Objective Function Index (COFI) method [36]. The COFI was computed as ${J}_{cr}$


(6) \begin{equation*} {J}_{\text{cr}}={J}_{\text{opt}}\cdot \left(1+\frac{p}{n-p}\cdot{F}_{\alpha, p,n-p}\right), \end{equation*}

where ${J}_{\text{opt}}$ denotes the minimum objective function value, *n* is the number of observations, *P* is the number of calibrated parameters, and ${F}_{{\alpha}, \text{p},\text{n}-\text{p}}$ denotes the value of the *F*-distribution, given *α* = 0.05 and the degree of freedom (*p*) and *n–p*. The feasible parameter space was determined by the parameters resulting in the total objective function values between ${J}_{\text{opt}}$ and ${J}_{\text{cr}}$.

The data assimilation generated two sets of best-fit parameters—one for the control and the other for the warming samples. We utilized the two sets of model parameters to conduct simulation experiments using 2010–2016 forcing data (soil temperature, soil moisture, and GPP). The resulting seasonal dynamics of intrinsic microbial CUE (referred to as the *Y*_g_ parameter in MEND) for the year 2012 were reported for both the warming and control conditions. Additionally, we also reported the temperature sensitivity of intrinsic microbial CUE, which was calculated as the slope of the linear regression between *Y*_g_ and temperature (known as the k*Y*_g_ parameter) for both the warming and control treatments.

The nonmicrobial Terrestrial ECOsystem model (TECO) is a CENTURY-type C pool and flux model, which simulates ecosystem C dynamics under various climatic conditions [38]. C dynamics in the TECO model can be described by a set of first-order ordinary differential equations, wherein turnover rates are modulated by soil temperature (T) and moisture (W) [39]. The prior ranges of transfer coefficients, turnover rates, and environmental scalars were determined based on previous studies [39, 40]. The posterior probability distributions of parameters were obtained using a Metropolis-Hastings (M-H) algorithm, a Markov Chain Monte Carlo (MCMC) technique, as previously described [39]. The TECO model simulated daily *R*_h_ for both warmed and control plots from 2010 to 2016. Consistent with the implementation of gMEND, we estimated the TECO model performance by calculating the coefficient of determination (*R*^2^) between the observed and simulated respiration.

## Results

### Changes in edaphic conditions, plants, and respiration by experimental warming

Here, we carried out a field study with experimental warming in a native tallgrass prairie ecosystem in Oklahoma, USA. We conducted monthly soil sampling (0–15 cm depth) and measurements in four replicates of warmed and control plots throughout the year of 2012 (the third year of experimental manipulation; Fig. S1). Our study site has a temperate climate characterized by sporadic drought, mild to warm summers, and cool to cold winters. The site has a 7-month growing season, typically from March to September. Based on both the growing season and local temperature variability (Fig. S3), we divided the year-round data into three seasons (i.e. the early cool season from January to February, the warm season from March to September, and the late cool season from October to December). This division is rooted in the recognition that microbial activity and plant phenology are closely tied not just to the static state of temperature but to the transitions and variability that define seasonal changes [41]. However, we did not combine the early and late cool seasons since the late cool season is directly impacted by plant litter from the warm season of the same year but the early season is impacted by plant litter from the preceding year, which means the soil microorganisms and how they handle C might not be the same in these two periods. Monthly mean soil temperature fluctuations were significant (*P* < 0.001) throughout the year (Table S1). The early cool season had an average background soil temperature of 6.5°C, and that of the warm season was 21.9°C. The late cool season had an average background soil temperature of 12.8°C.

Global warming was simulated by heating the air and soils with infrared heaters, which increased the average annual soil temperature by 4.3°C (*P* < 0.001; Table S1 and Fig. S4). Specifically, warming increased the average soil temperature by 4.5°C in the warm season, higher than those in the early cool season (3.6°C) and the late cool season (4.1°C). This difference could be attributed to heat loss in the cool seasons derived from strong winds and lower ambient temperature. Due to the high water-holding capacity of plants, the warming treatment did not alter soil moisture in the warm season, but significantly reduced soil moisture during the early cool season (from 10.7% to 5.9%) and the late cool season (from 7.5% to 5.3%). Although the soil NH_4_^+^ content was similar across those plots (Table S1), the NO_3_^−^ content was considerably higher in warmed plots than control plots (an average of 6.29 mg/kg in control plots and an average of 10.77 mg/kg in warmed plots, *P* = 0.001). The increase in NO_3_^−^ content by warming was mainly observed in the late cool season (Table S1).

**Figure 1:**
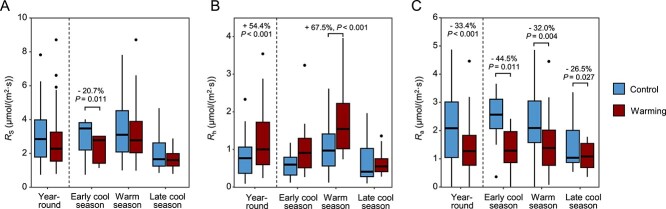
Temporal changes of soil respiration and its components under warming and control. (A) Soil respiration (*R*_S_). (B) Soil heterotrophic respiration (*R*_h_). (C) Soil autotrophic respiration (*R*_a_). The data are segmented into the early cool season (January to February), warm season (March to September), and late cool season (October to December), and comparisons are made across these periods as well as throughout the year. Significant changes in respiration rates under warming compared to control conditions are indicated, with statistical analyses performed using paired *t*-tests. All exact values and *P-*values, for both significant and nonsignificant changes, are detailed in Table S1.

Warming increased *R*_h_ by 54.4% throughout the year, while reducing *R*_a_ by 33.4% (*P* < 0.001, Table S1 and Fig. 1). As a result, *R*_S_ remained unchanged by warming due to an offset between the increasing *R*_h_ and decreasing *R*_a_ (*P* > 0.050, Table S1 and Fig. 1). In addition, no significant warming effect was observed for ecosystem respiration (*R*_e_), net ecosystem exchange (NEE), or gross primary production (GPP) (*P* > 0.050; Table S1). In addition, almost all soil climatic, physiochemical, plant, and respiration variables varied across months (*P* < 0.050, Fig. S4), except for total nitrogen (N) content. In warmed plots, we observed early autumn peaks of GPP and *R*_e_ (Fig. S4H and J). The warming treatment also lowered spring peaks of GPP and *R*_e_ in March, suggesting that warming could impose stress on plant communities in temperate grasslands, resulting in inhibited plant growth and productivity.

### Microbial functional traits at the gene level

Soil ecosystem functioning is closely related to microbial functional traits, such as genes or enzymes, which are often decoupled from taxonomy [42]. Here, we employed a microarray-based tool named GeoChip 5.0 to analyze 1010 key functional genes associated with C, N, and phosphorus cycling, electron transfer, and organic remediation [32]. Microbial community functional compositions were markedly different between warmed and control samples and among months, which were verified by all three different complementary nonparametric multivariate statistical tests (Adonis, ANOSIM, and MRPP; Table S2). There was a significant interactive effect between warming and time (*P* = 0.001 for Warming × Month) on microbial functional composition, unveiling the season-dependent changes in the abundances of certain genes and possibly the microorganisms that host them.

**Figure 2:**
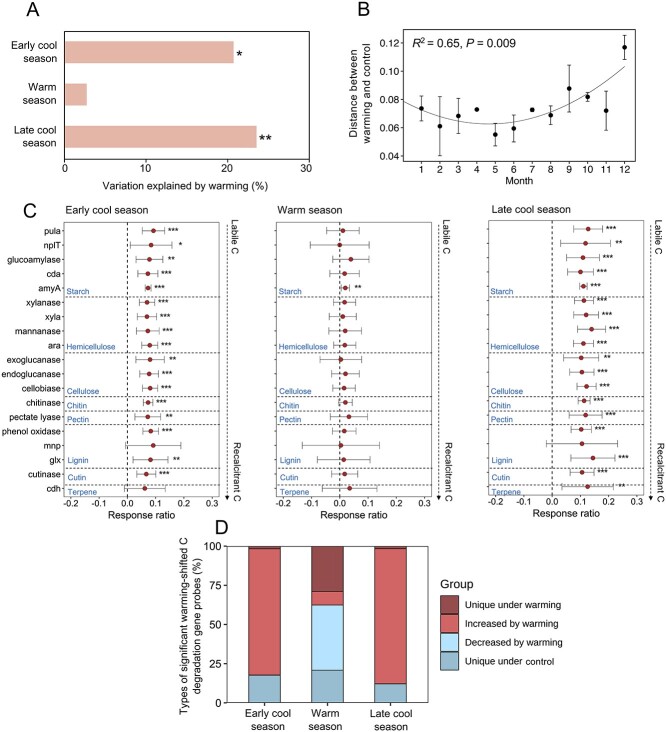
Warming effects on microbial functional composition and C-decomposing genes. (A) the percentage of variation in microbial functional compositions explained by warming in the early cool season (January to February), warm season (March to September), and late cool season (October to December), as tested by Adonis. Significances are indicated by ** when *P* < 0.010 and * when *P* < 0.050. (B) Dissimilarities of microbial functional compositions between warming and control on a monthly basis. The dissimilarity values of paired warmed and control samples were fitted to nonlinear quadratic regression. The *R*^2^ and *P-*values were calculated, reflecting the variance explained by the regression. Distances were calculated based on the Bray-Curtis metric. (C) Response ratios showing changes in the abundance of functional genes associated with C decomposition between warmed and control samples in the early cool season (January to February), warm season (March to September), and late cool season (October to December). C substrates are arranged in the order from labile C to recalcitrant C. Error bars indicate 95% confidence intervals of abundance differences between warmed and control samples. (D) Percentages of significant changes of C-decomposing gene probes by warming. Probes are classified into four categories: unique under warming (probes detected only in warmed samples, and those that were likely present in control samples but below the level of detection), increased under warming (the response ratio > 0, *P* < 0.050), increased under warming (the response ratio > 0, *P* < 0.050), and unique under control (probes detected only in control samples, and those that were likely present in warmed samples but below the level of detection).

Warming primarily affected overall microbial functional compositions during cool seasons, explaining 20.8% to 23.5% of compositional variations (Fig. 2A). In sharp contrast, the warming effect was negligible and insignificant during the warm season. We then determined the differences in microbial functional compositions between paired warmed and control plots on a monthly basis (Fig. 2B). The differences exhibited a nonlinear, U-shaped relationship (*R*^2^ = 0.65, *P* = 0.009), with the largest differences observed between warming and control during the cool months. The relative abundances of genes associated with C decomposition, measured by GeoChip, were either increased or unchanged by warming, with the largest increases observed in the cool seasons (the response ratio analysis at the 95% confidence interval, Fig. 2C). Examples of increased genes include *amyA* encoding amylase that hydrolyzes starch and glycogen, *xylanase* that hydrolyzes hemicellulose, *cellobiase* that hydrolyzes cellobiose to glucose, *chitinase* that degrades chitin, *pectinase* that degrades pectic substances, glyoxal oxidase (*glx*) associated with lignin decomposition, and *cutinase* associated with cutin decomposition. Relative abundances of almost all genes were increased (*P* < 0.050) in the cool seasons, except for a few genes encoding recalcitrant C decomposition (e.g. *mnp* associated with lignin decomposition). Conversely, only one gene (i.e. *amyA*) was increased (*P* < 0.050) in relative abundance by warming in the warm season, whereas others remained unchanged (Fig. 2C).

We examined functional genes at the probe level, as GeoChip contains multiple oligonucleotide probes for detecting sequence variants of the same functional gene [32]. A total of 61 569 microbial functional gene probes were detected. The seasonal dependence of warming-induced stimulation of microbial C-decomposing gene abundances was still evident, as the percentages of significant warming-stimulated (i.e. response ratio > 0, *P* < 0.050) gene probes were much higher in the cool seasons (80.5% in the early cool season and 86.1% in the late cool season) than in the warm season (8.7%) (Fig. 2D and Table S3).

To determine how changes in microbial carbon-decomposing traits by warming affect *R*_S_, we performed partial Mantel tests to link the relative abundances of microbial carbon-decomposing genes with *R*_S_, controlling for the effect of other confounding variables (Table S4). Most C-decomposing genes in both warmed and control plots were correlated with *R*_h_ and *R*_S_ (*P* < 0.050) but not *R*_a_ (*P* > 0.400). Larger correlation coefficients were found between C-decomposing genes and *R*_h_ than *R*_S_ (*r* = 0.41–0.61, *P* < 0.013 for *R*_h_; *r* = 0.38–0.46, *P* < 0.022 for *R*_S_).

### Microbial functional traits at the community level

The average rRNA operon (*rrn*) copy number is a community-level functional trait that reflects microbial life strategy, as it correlates with maximal growth rate in response to resource availability [43]. To estimate community *rrn* copy numbers, we carried out 16S ribosomal RNA gene-based amplicon sequencing to profile microbial taxonomic compositions, obtaining a total of 44 571 amplicon sequence variants (ASVs) for 96 samples after randomly resampling 16S rRNA gene sequences to the same depth (21 567 sequences per sample, the minimum sequence count across all samples). Similar to observations in microbial functional compositions, significant treatment, month, and interactive effects were detected for microbial taxonomic compositions, with substantial seasonal variations surpassing those of warming (Table S2). Variations in microbial taxonomic compositions explained by warming were much greater in cool seasons than warm season (Fig. S5), suggesting that the magnitudes of warming effects on microbial taxonomic compositions were more significant in cool seasons.

Warming affected the relative abundances of soil microbial taxa (Fig. S6A). Specifically, warming increased (*P* < 0.050) the relative abundances of *Firmicutes*, but decreased those of *Actinobacteriota*, *Planctomycetota, and Myxococcota* across all months (Fig. S6B). During early and late cool seasons, we observed decreases in relative abundances of *Acidobacteriota* and *Planctomycetota* (Fig. S6C and E). In contrast, in the warmer season, we observed a decrease in the relative abundances of *γ-Proteobacteria*, *Bacteroidota*, and *Planctomycetota*, along with an increase in those of *Actinobacteriota* and *Chloroflexi* (Fig. S6D).

**Figure 3:**
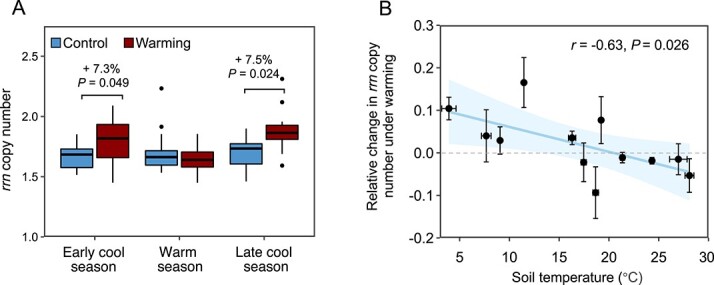
Warming effects on the community-level functional traits of microbial communities. (A) The average *rrn* copy number, calculated as the sum of the abundance weighted *rrn* copy number of each ASV in a sample and averaged in the early cool season (January to February), warm season (March to September), and late cool season (October to December). Error bars represent standard errors. The differences between the control and warming treatment were tested with paired *t*-tests. (B) Correlation between soil temperature and relative change in community average *rrn* copy number by warming. Soil temperatures were measured in control plots. Relative changes in community average *rrn* copy number by warming were calculated as the logarithmic differences between average *rrn* copy numbers of paired samples between warmed and control plots. Bidirectional error bars denote standard errors. The dashed horizontal line represents the relative change in *rrn* copy number equals 0 (i.e. *rrn* copy number unchanged by warming). Pearson correlation coefficient (*r*) and *P-*value are shown.

We calculated the average *rrn* copy number of each bacterial community and found that it was increased by warming only during cool seasons (Fig. 3A), despite large monthly variations (*P* = 0.005; Fig. S7A). There was a negative correlation (*r* = −0.63, *P* = 0.026) between the relative change in the average *rrn* copy number under warming and background soil temperature (Fig. 3B), suggesting that the warming effect on the average *rrn* copy number was proportional to temperature anomalies. However, the average *rrn* copy number decreased with soil temperature only in warmed plots, but not in control plots (Fig. S7B), implying that microbial communities under warming were more sensitive to rapid, local temperature variability. In addition, changes in average *rrn* copy number were positively correlated with those of C-decomposing gene abundances at marginal significance (*r* = 0.55, *P* = 0.067, Fig. S7C), highlighting a linkage between gene-based functional capability and a community-aggregated functional trait.

The community-level microbial metabolic quotient, defined as *R*_h_ per unit of microbial biomass, is a crucial determinant of soil C turnover [44]. Soil microbial biomass, represented by DNA yield, remained unchanged by warming (*P* > 0.050, Fig. S8A and B) despite significant monthly variations (*P* = 0.003, Fig. S8A). Microbial biomass peaked in April and October, trailing one month behind the peaks of plant GPP, showing a delayed response of belowground communities to changes in aboveground ecosystems. We found marked monthly variations in microbial metabolic quotients (*P* < 0.001, Fig. S8C). Warming increased microbial metabolic quotients (*P* < 0.001), and its effect varied with the time of year (*P* = 0.081 for warming × month interaction). The warming-induced increase in microbial metabolic quotient was most prominent during cool seasons, with a 113.4% increase in the early cool season (*P* = 0.046) and a 69.1% increase (*P* = 0.006) in the late cool season (Fig. S8D). In contrast, the magnitude of microbial metabolic quotient was only increased by 37.3% during the warm season (*P* = 0.019), revealing a lower sensitivity of soil microbial community and soil C to warming.

### Ecosystem model evaluation

Ecologists face a formidable challenge in integrating microbial community information, especially omics data, into ecosystem models [45]. Our previous efforts demonstrated that the gMEND model, which was developed by integrating functional gene abundance into an ecosystem model named the Microbial-ENzyme Decomposition Model (tMEND) could improve parameterization and the model’s performance [18]. In this study, we used monthly gene abundance data to calibrate the gMEND model, alongside other input data such as soil temperature, soil moisture, GPP, and *R*_h_. The MEND model explicitly represents microbial physiology and soil organic matter (SOM) decomposition catalyzed by oxidative or hydrolytic enzymes. Given that the model necessitates absolute quantitative data on these enzymes for SOM decomposition, we utilized GeoChip-detected abundances of corresponding functional gene data. We constrained gMEND by achieving the highest correlation between the modeled enzyme concentrations and GeoChip-detected oxidative and hydrolytic gene abundances, while simultaneously attaining the best fit between observed and simulated *R*_h_. We observed strong correlations between simulated enzyme concentrations and GeoChip-detected gene abundances under both control (*r* = 0.56 for oxidative enzymes and *r* = 0.64 for hydrolytic enzymes) and warming (*r* = 0.76 for oxidative enzymes and *r* = 0.86 for hydrolytic enzymes, Fig. S9A–D). This indicated good agreements on the monthly variabilities between simulated enzyme concentrations and GeoChip-detected gene abundances.

**Figure 4:**
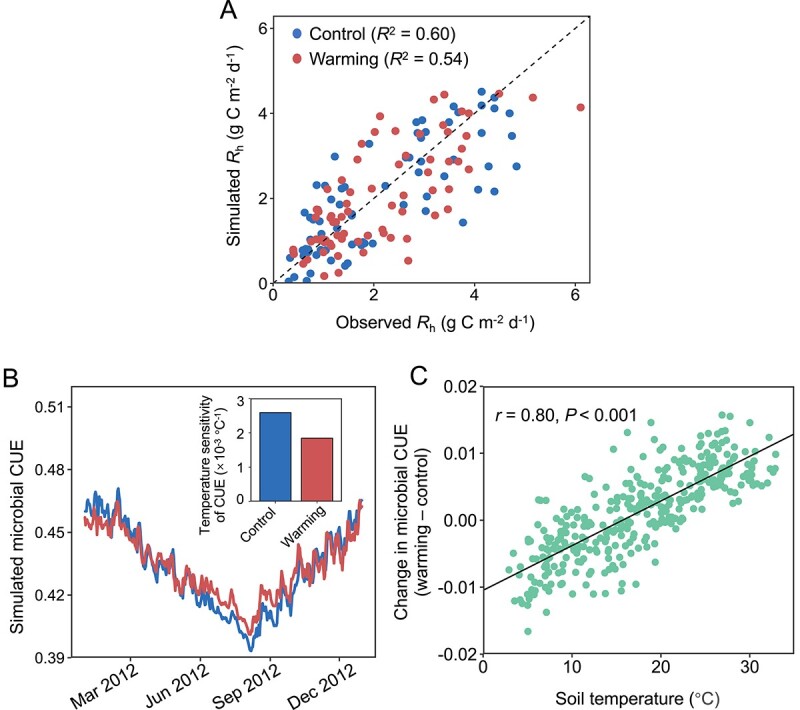
Model performance and model-derived microbial C use efficiency (CUE) changes. (A) Comparison between gMEND-simulated and observed *R*_h_ under warming and control (*R*^2^ denotes the coefficient of determination). (B) Simulated daily microbial CUE in 2012, as determined by the estimated C use efficiency (*Y*_g_(*T*_ref_)), the slope for *Y*_g_ dependence on temperature, and soil temperature. The inset graph shows the modeled temperature sensitivity of microbial CUE (i.e. the slope for *Y*_g_ dependence on temperature) under warming and control. (C) The difference in microbial CUE between warming and control samples as a function of soil temperature. Soil temperatures shown here are soil temperatures in the control plots. Pearson correlation coefficient (*r*) and *P-*value are shown.

The gMEND-simulated *R*_h_ agreed well with observed *R*_h_ (*R*^2^ = 0.54 for warming samples and 0.60 for control samples, Fig. 4A). Moreover, gMEND outperformed tMEND in constraining model parameters under both warming and control, resulting in a significant reduction in the average coefficient of variation (CV) of model parameters (−19.8% under control and − 29.7% under warming, Fig. S9E). gMEND also improved *R*_h_ flux fitting by 7.9% under control and by 20.7% under warming compared to a widely used terrestrial ecosystem model named TECO without microbial parameters (Fig. S9F and G).

Microbial CUE determines the partitioning of substrate carbon between microbial biomass and CO_2_ production. Because we did not experimentally measure CUE, we estimated it using parameter *Y*_g_ in the best model parameter sets under warming and control. The estimated CUE ranged between 0.39 and 0.47 (Fig. 4B). However, its “apparent” temperature sensitivity, which incorporated both direct and indirect environmental influences on microbial CUE [45], was reduced by warming (Fig. 4B, inset). As a result, the difference in CUE between warmed and control plots switched from being negative to being positive at ~15.7°C, and was strongly and positively correlated with ambient temperature (*r* = 0.80, *P* < 0.001, Fig. 4C).

### Explaining *R*_h_ and *R*_S_ dynamics from a trait perspective

**Figure 5:**
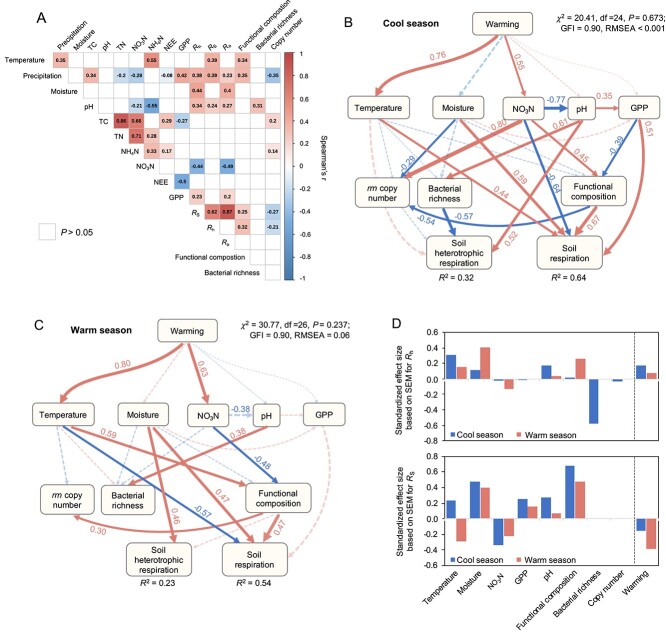
Relationships among environmental variables, microbial communities, and respiration. (A) Spearman correlations among environmental variables (temperature, precipitation, pH, soil moisture, NO_3_^−^, and total C), microbial functional traits (functional composition and community average *rrn* copy number), gross primary productivity (GPP), *R*_h_ and *R*_S_. (B and C) Structural equation models (SEM) to assess the relationships among environmental variables, microbial functional traits, gross primary productivity (GPP), *R*_h_ and *R*_S_ in (B) the cool season and (C) the warm season. Solid lines represent significant direct effects (*P* < 0.050), while dashed lines represent insignificant paths. Numbers adjacent to the arrow are the standardized path coefficients (for significant pathways only), and arrow width indicates the proportional strength of the pathway. *R*^2^ indicates the proportion of variations explained for the dependent variable in the model. The goodness-of-fit of the SEM was estimated by the chi-square (*χ*^2^) test and root mean square error of approximation (RMSEA). (D) Standardized total effects (direct plus indirect effects) derived from SEMs.

*R*_S_ was positively correlated with soil moisture (*r* = 0.44), gross primary productivity (GPP, *r* = 0.34), and soil pH (*r* = 0.34), but negatively correlated with NO_3_^−^ content (Fig. 5A). Microbial functional composition showed positive associations with both *R*_S_ (*r* = 0.25) and *R*_h_ (*r* = 0.32). Soil temperature (*r* = 0.39), precipitation (*r* = 0.39), and soil pH (*r* = 0.24) were also positively associated with *R*_h_. To further understand the mechanisms behind these correlations and to discriminate the direct and indirect effects of warming on *R*_h_ and *R*_S_, environmental variables, and microbial taxonomic and functional traits (community richness, *rrn* copy number, and microbial genes comprising functional compositions), we performed structural equation modeling (SEM) for both the cool (Fig. 5B) and warm seasons (Fig. 5C). In the cool season, warming directly increased soil temperature (β = 0.76, *P* < 0.001) and NO_3_^−^ content (β = 0.55, *P* = 0.001) (Fig. 5B). A positive linkage was observed between microbial community *rrn* copy number and soil NO_3_^−^ content (β = 0.80, *P* < 0.001), and a negative linkage with soil moisture (β = −0.29, *P* = 0.045). In the warm season, although warming similarly affected soil temperature and NO_3_^−^ content, the associations between environmental factors and microbial traits were less significant and generally weaker (Fig. 5C). *R*_h_ was directly influenced by bacterial richness (β = −0.57, *P* = 0.007) and soil pH (β = 0.52, *P* = 0.014) in the cool season, but directly influenced by soil moisture (β = 0.46, *P* = 0.001) in the warm season. *R*_S_ was directly affected by an array of biotic and abiotic factors, including soil temperature, moisture, NO_3_^−^ content, microbial functional composition, and GPP (Fig. 5B and C). Among them, *R*_S_ exhibited a strong positive correlation with GPP in the cool season (β = 0.51, *P* < 0.001), a relationship that was not evident in the warm season (*P* = 0.121). Additionally, the *R*_S_ linkage with microbial functional compositions was stronger in the cool season than in the warm season (β = 0.67 vs 0.47). Accordingly, the SEMs explained a greater proportion of the variation in *R*_h_ and *R*_S_ to warming in the cool season (31.9% for *R*_h_, 64.1% for *R*_S_, Fig. 5B) compared to the warm season (23.1% for *R*_h_, 53.6% for *R*_S_, Fig. 5C).

SEM analysis revealed that warming played a more predominant role in stimulating *R*_h_ in the cool season than in the warm season (standardized total coefficient = 0.17 vs 0.07; Fig. 5D) through both direct and indirect pathways. In contrast, *R*_S_ in warm months was more shaped by warming-induced changes compared to that in the cool months (standardized total coefficient = −0.16 vs −0.39; Fig. 5D).

## Discussion

### Warming-induced changes in edaphic conditions, plants, and respiration

The increased *R*_h_ by warming throughout the year (Fig. 1) was consistent with the predictions from the Metabolic Theory of Ecology [46] and a recent analysis showing that global *R*_h_ has increased steadily since 1987 [47]. In contrast, *R*_a_ decreased with warming across all seasons (Fig. 1). However, the underlying mechanisms driving decreased *R*_a_ may vary. In cooler seasons, such as the early and late cool seasons, the decrease in *R*_a_ in response to warming could be attributed to warming-induced water stress, as evidenced by significant reductions in soil moisture of 45.1% in the early cool season and 29.4% in the late cool season (Table S1). Plant growth processes including photosynthesis, respiration, and transpiration have been shown to have parabolic temperature response curves with ecosystem-dependent optimal temperature [48]. During the warm season, high ambient soil temperature may be the reason that limits plant growth and *R*_a_, as warming lowered the spring and autumn peaks of *R*_e_ and GPP (Fig. S4), in addition to the early autumn peaks of GPP and *R*_e_ in warmed plots (Fig. S4H, J) that were consistent with the previous finding that climate warming affects autumn senescence [49]. The elevated soil temperatures associated with warming scenarios could exacerbate heat stress, leading to further declines in *R*_a_. The disparity in responses between *R*_h_ and *R*_a_ to warming suggests the involvement of distinct mechanisms in microbial and plant communities. The increase in *R*_h_ by warming likely indicates heightened soil C mineralization and subsequent C loss facilitated by microbial decomposition.

### Microbial functional traits

GeoChip has been successfully used in a wide range of habitats [50–52]. Direct comparisons between GeoChip and metagenomics shotgun sequencing technologies have consistently yielded similar results [32, 52]. GeoChip offers a distinct advantage in quantitative measurements, displaying comparable accuracy to real-time PCR and higher accuracy than shotgun sequencing [53, 54].

Compared to the warm season, the higher percentages of significant warming-stimulated gene probes in the cool seasons (Fig. 2D and Table S3) agree with previous studies conducted at both local and global scales showing that the abundances of microbial C-decomposing genes responded positively to warming in soils from cold climates [52, 55]. It suggests that the climatological temperature control on C turnover is more sensitive in soils from cold climates than those in warm climates, as observed previously [56]. Due to close linkages between C-decomposing genes and *R*_S_ (Table S4), warming can potentially accelerate soil C loss derived from microbial decomposition. As a result, soil C pools may be particularly vulnerable to warming during cool seasons.

Similar to observations in microbial functional compositions, significant treatment, month, and interactive effects were detected for microbial taxonomic compositions, with substantial seasonal variations surpassing those of warming (Table S2). These results were concordant with previous studies showing that the effects of climate change treatments, e.g. warming [10, 57, 58], intensified precipitation [10], drought, nitrogen addition, and their interactions [9] on microbial communities were subtle compared with pronounced seasonal patterns, and that warming effects varied substantially with sampling time.

Warming might create more or alternative niche space [59, 60], eliciting the selection of microorganisms with particular traits that translate into life strategies. During early and late cool seasons, there were decreases in relative abundances of *Acidobacteriota* and *Planctomycetota* (Fig. S6C and E), which are typical oligotrophs characterized by low *rrn* copy numbers [61, 62]. Similarly, experimental warming decreased the relative abundance of *Acidobacteriota* in the continuous permafrost region of Northeastern China [63]. The relative abundance of *γ-Proteobacteria*, generally regarded as copiotrophic and characterized by high *rrn* copy numbers [64], was decreased in the warmer season (Fig. S6D). In contrast, the relative abundance of *Actinobacteriota* a typical oligotrophic phylum [65], was increased (Fig. S6D), which was also observed in other ecosystems [63].

The average *rrn* copy number decreased with soil temperature only in warmed plots, but not in control plots (Fig. S7B), likely owing to diversity loss [8] and higher species turnover rate [66] elicited by warming. This finding was consistent with previous studies in forest soils that revealed negative correlations between community *rrn* copy number and temperature [59, 67]. In addition, changes in average *rrn* copy number were positively correlated with those of C-decomposing gene abundances (Fig. S7C), indicating that warming increased the number of *rrn* copies in microorganisms with higher abundances of C-decomposition genes.

The warming-induced increase in microbial metabolic quotient was most prominent during cool seasons (Fig. S8D). Consistently, a positive relationship between microbial metabolic quotient and soil temperature was theoretically predicted and experimentally observed [68]. Multiple mechanisms contribute to the warming effect because warming accelerates protein turnover and microbial metabolic activity [69], releases C more rapidly from soil microorganisms by increasing the activity of microbial predators and bacteriophages [70], and shifts microbial community composition towards fast-growing species as observed in our study (Fig. 3A).

### Ecosystem model and SEM analyses

The estimated CUE ranged between 0.39 and 0.47 (Fig. 4B), which aligned closely with soil microbial CUE measurements under in situ conditions using ^18^O isotope tracers or through stoichiometric modeling [71–73]. Microbial CUE was lower in warmed plots than control plots under soil temperatures of 15.7°C (Fig. 4C), which fell within the range of average soil temperature during the cool seasons. The lower CUE under warming in cool seasons corresponds with higher community average *rrn* copy number (Fig. 3A) and relative abundances of C-decomposing genes (Fig. 2C), in support of the growth rate–efficiency tradeoff in heterotrophic metabolism [74]. Our finding that warming led to a decrease in microbial CUE during cooler months, echoing theories of elevated temperatures boosting metabolic energy needs and thus reducing CUE [71, 75]. In contrast, winter warming increased the microbial CUE in subarctic regions [76]. This discrepancy may arise from substantial increases in the number and intensity of freeze–thaw cycles induced by winter warming in subarctic areas, which selected for a more resilient community with higher CUE and growth rate [76]. These variable responses underscore the critical role of local environments in shaping microbial adaptation to climate change.

SEM analysis revealed that microbial community *rrn* copy number was positively linked to soil NO_3_^−^ content but negatively linked to soil moisture (Fig. 5B), suggesting microbial adjustment in response to dynamic moisture and resource conditions [43]. The warming effects on *R*_h_ and *R*_S_ were distinct (Fig. 5D), which could be attributed to the contrasting responses of *R*_a_ and *R*_h_ to warming. Due to the infrequent and highly variable fine-scale measurements of *R*_h_, our findings contribute to the ongoing discussion regarding its temperature sensitivity, demonstrating that *R*_h_ is likely more responsive to warming during cooler months. This response could amplify the positive feedback loop between soil carbon dynamics and the atmosphere. Our results also support the idea that current *R*_h_ and *R*_S_ models based on fixed parameters (e.g. the fixed temperature in an exponential function) are inadequate for describing the respiration response. Without accounting for higher temperature sensitivity in cool months, Earth system models will likely underestimate *R*_h_ rates provoked by warming, particularly in those periods.

Our observations lend support to the growing body of evidence that microbial activity and associated functional traits persist and may even intensify during the cooler months, despite traditionally being considered periods of reduced biological activity [77–79]. The enhanced temperature sensitivity of soil *R*_h_ observed in our study is unlikely to be caused by temperature-specific differences, as warming increased the average soil temperature more in the warm season than in the cool seasons (Table S1 and Fig. S4). Instead, the significant reductions in soil moisture during the early and late cool seasons, combined with increased NO_3_^−^ content in the late cool season, suggest that these factors substantially contribute to the observed seasonal patterns in microbial and ecosystem responses to warming [37]. Climate-mediated increases in nutrient availability for soil microbes were particularly evident in conjunction with the soil freezing–thawing cycles [80, 81]. In addition, the presence of a distinctive microbiome capable of rapid growth and rapid substrate utilization even under cold soil temperature regimes could also be attributable [82]. Our comprehensive, multifaceted approach delineates the taxonomic and functional attributes of an active and responsive soil microbiome, concurrently presenting data that connect nutrient dynamics to *R*_h_ variability. By synthesizing time-series analyses with a functional trait-based framework, our research critically advances the understanding of microbial activity in response to climatic warming. The integration of functional gene data into ecosystem models propels our comprehension of microbial functional potential, thus considerably enhancing the predictability of ecological responses to climate change. Such methodological innovation in incorporating microbial dynamics bridges a gap not fully explored by prior studies and stands to significantly influence future ecosystem modeling endeavors.

## Conclusion

Here, our time-series analyses of respiration, microbial life strategy, metabolic quotient, C-decomposing potential, and CUE suggest greater sensitivities of *R*_h_ and *R*_S_ to experimental warming in the cool season than in the warm season. Despite that we observed no changes in total soil C stock since it is a vast reservoir that buffers short-term fluctuations, it is likely that the detected changes in *R*_h_ and *R*_S_ will eventually affect the long-term balance of soil C, especially as such periods extend or intensify with ongoing global warming.

Warming increased the average community *rrn* copy number and the relative abundances of functional genes involved in C degradation in the cool seasons, linking microbial functional traits to *R*_h_ and *R*_S_. To accurately predict ecosystem responses to climate warming, our study identified key controls of microbial functional traits, going beyond temperature, to be considered in Earth system models. Our findings also challenge the common practice of analyzing soil microbial communities only during plant growth season by emphasizing the importance and unique characteristics of soil microbial communities in the cool season.

## Supplementary Material

Microbial_Traits_Soil_Warming_Supporting_Info_wrae088(1)

## Data Availability

DNA sequences of 16S rRNA gene amplicons are available in NCBI Sequence Read Archive under project no. PRJNA626428. GeoChip data are available online (www.ncbi.nlm.nih.gov/geo/) with the accession number GSE195490.

## Code availability

The data and R code that support the findings of this study are openly available at https://github.com/jiesi-lei/monthly-soil-microbiome.
