# Supplementary material for: Warming effects on grassland soil microbial communities are amplified in cool months
Source: ISME J. 2024 May 15;18(1):wrae088. doi: 10.1093/ismejo/wrae088 (PMC11170927; doi:10.1093/ismejo/wrae088)
Supplement: Microbial_Traits_Soil_Warming_Supporting_Info_wrae088(1) [file microbial_traits_soil_warming_supporting_info_wrae088(1).pdf]

Supplementary Information

Supplementary Table S1. Summary of environmental variables in different seasons

Variables	Early cool season		Warm season		Late cool season		All year	
	Control	Warming	Control	Warming	Control	Warming	Control	Warming
Soil temperature (°C)	6.49a±1.03[a]	10.04b±0.78	21.88a±0.85	26.37b±0.93	12.78a±1.46	16.85b±1.67	17.04a±1.08	21.27b±1.16
Soil moisture (%)	10.69a±0.81	5.87b±0.83	8.56±0.76	7.82±0.68	7.52a±0.79	5.31b±0.92	8.65a±0.52	6.87b±0.50
pH	6.36±0.12	6.60±0.09	6.42±0.05	6.46±0.06	6.25±6.09	6.09±0.09	6.36±0.04	6.39±0.05
Total C (%)	0.82±0.09	0.66±0.06	0.87±0.06	0.81±0.05	0.92±0.09	1.06±0.10	0.88±0.04	0.85±0.04
NO_3N (mg/kg)	1.85±0.67	1.32±0.13	7.96±1.41	10.72±1.66	5.36a±1.15	17.20b±3.37	6.29a±0.93	10.77b±1.46
NH_4N (mg/kg)	11.39±0.83	10.17±0.75	11.23±0.53	11.07±0.49	12.12±0.67	12.93±1.04	11.48±0.37	11.39±0.42
Total N (%)	0.08±0.01	0.07±0.01	0.08±0.01	0.08±0.004	0.10±0.02	0.10±0.01	0.09±0.006	0.08±0.004
GPP (μmol/(m^2·s))	4.53±0.82	4.61±0.51	6.80±1.24	5.08±1.06	2.46±0.51	1.92±0.56	-5.34±0.78	-4.21±0.66
NEE (μmol/(m^2·s))	-2.45±0.51	-2.38±0.34	-1.47±0.53	-1.15±0.46	-0.81±0.21	-0.63±0.21	-1.47±0.33	-1.23±0.29
R_e (μmol/(m^2·s))	2.08±0.45	2.23±0.33	5.33±0.92	3.93±0.75	1.65±0.62	1.30±0.51	3.87±0.61	2.98±0.48
R_S[b] (μmol/(m^2·s))	2.92a±0.43	2.31b±0.30	3.40±0.31	3.23±0.34	2.11±0.35	1.65±0.19	3.0±0.22	2.68±0.23
R_h (μmol/(m^2·s))	0.52±0.12	1.02±0.30	0.92a±0.10	1.54b±0.14	0.66±0.17	0.58±0.09	0.79a±0.08	1.22b±0.11
R_a (μmol/(m^2·s))	2.40a±0.37	1.33b±0.28	2.48a±0.27	1.69b±0.26	1.45a±0.25	1.07b±0.14	2.21a±0.19	1.47b±0.17
Precipitation(mm)	32.00±1.15	32.00±1.15	81.21±7.38	81.21±7.38	15.16±2.22	15.16±2.22	56.49±6.11	56.49±6.11

[a]All data are presented as mean ± s.e. calculated from replicates measured on a monthly basis. Differences between control and warming groups in each season and throughout the year were tested with Student's *t*-test, with significant differences marked by different letters. The detailed information of these measurements and the significance of warming treatment, sampling month, or their interaction are summarized in Fig. S1.

[b]R_S and R_h were measured directly in the field, and R_a was calculated as the difference between R_S and R_h. Due to the inherent variability in soil CO_2 efflux, R_S exceeded R_h in two samples, leading to negative values for R_a. During data cleaning, we adjusted negative R_a values to zero while retaining the original measurements for R_h and R_S.

Supplementary Table S2. Main and interactive effects of warming and months on the overall microbial taxonomic and functional composition based on permutation multivariate analysis of variance (Adonis)[a]

		Taxonomic			Functional		
		Warming	Month	Warming × Month	Warming	Month	Warming × Month
Adonis[a]	*F*	3.3	1.6	1.0	24.4	43.1	5.7
	R^2	0.030	0.161	0.098	0.038	0.736	0.097
	p	**0.001**[b]	**0.001**	**0.001**	**0.001**	**0.001**	**0.001**
MRPP	δ	1396	1350	N.A.[c]	580.5	397.5	N.A.
	p	0.07*	0.002	N.A.	**0.001**	**0.001**	N.A.
ANOSIM	*R*	0.094	0.117	N.A.	0.019	0.637	N.A.
	p	**0.001**	**0.001**	N.A.	**0.094**	**0.001**	N.A.

[a]Adonis, Permutation multivariate analysis of variance; MRPP, Multiple response permutation procedure; ANOSIM, Analysis of similarity. Bray-Curtis distances were used for dissimilarities tests.

[b]Significant differences with *p* values less than 0.050 are marked in bold.

[c]N.A., not applicable

Supplementary Table S3. Microbial C-decomposing gene probes significantly shifted by warming in different seasons

Types	Early cool season	Warm season	Late cool season
Unique under warming[a]	39 (1.7%)[b]	183 (28.9%)	65 (1.7%)
Increased under warming	1828 (80.5%)	55 (8.7%)	3324 (86.1%)
Decreased under warming	3 (0.1%)	263 (41.6%)	5 (0.3%)
Unique under control	401 (17.7%)	132 (20.9%)	465 (12.1%)
Total	2271 (100.0%)	633 (100.0%)	3859 (100.0%)

[a]C-decomposing gene probes with significant warming-induced changes in relative abundances under warming are classified into four categories: unique under warming (probes detected only in warmed plots, and those likely present in the early and late cool seasons but below the level of detection), increased under warming (response ratio > 0, $p < 0.050$), unique under unwarming (probes detected only in control plots, and those likely present in the warm season but below the level of detection).

[b]The data are presented as counts (percentage).

Supplementary Table S4. Partial Mantel tests of microbial C-decomposing gene composition and R_S[a]

Gene[b]	R_S				R_h				R_a			
	Control		Warming		Control		Warming		Control		Warming	
	r	*p*	*r*	*p*	*r*	*p*	*r*	*p*	*r*	*p*	*r*	*p*
amyA	0.36	**0.008**[c]	0.41	**0.013**	0.31	**0.042**	0.41	**0.013**	0.02	0.405	-0.05	0.517
cda	0.37	**0.011**	0.40	**0.013**	0.31	**0.030**	0.53	**0.003**	0.02	0.423	-0.05	0.532
glucoamylase	0.35	**0.017**	0.43	**0.006**	0.32	**0.021**	0.56	**0.003**	<0.01	0.477	-0.04	0.442
nplT	0.46	**0.002**	0.44	**0.011**	0.32	**0.033**	0.57	**0.003**	0.09	0.272	-0.01	0.381
pula	0.41	**0.006**	0.41	**0.007**	0.33	**0.016**	0.56	**0.004**	0.04	0.374	-0.03	0.448
ara	0.40	**0.007**	0.46	**0.003**	0.31	**0.035**	0.60	**0.002**	0.04	0.371	-0.04	0.476
mannanase	0.39	**0.007**	0.40	**0.008**	0.28	**0.045**	0.57	**0.003**	0.04	0.375	-0.05	0.550
xylA	0.37	**0.007**	0.42	**0.007**	0.31	**0.034**	0.57	**0.002**	0.03	0.406	-0.06	0.533
xylanase	0.33	**0.032**	0.43	**0.005**	0.29	0.074	0.61	**0.002**	-0.01	0.469	-0.04	0.471
cellobiase	0.41	**0.005**	0.42	**0.008**	0.31	0.056	0.54	**0.006**	0.06	0.355	-0.01	0.408
endoglucanase	0.36	**0.024**	0.42	**0.019**	0.29	0.078	0.59	**0.001**	0.02	0.382	-0.05	0.521
exoglucanase	0.36	**0.012**	0.41	**0.012**	0.29	**0.043**	0.61	**0.001**	0.01	0.438	-0.06	0.572
chitinase	0.35	**0.009**	0.40	**0.008**	0.28	**0.044**	0.57	**0.001**	0.02	0.421	-0.06	0.536
fungi chitin deacetylase	0.39	**0.007**	0.41	**0.010**	0.30	0.059	0.57	**0.001**	0.04	0.374	-0.05	0.501
pectate lyase	0.42	**0.004**	0.39	**0.022**	0.36	**0.006**	0.58	**0.003**	0.04	0.392	-0.06	0.541
glx	0.38	**0.005**	0.38	**0.013**	0.24	0.056	0.51	**0.005**	0.04	0.403	-0.08	0.642
mnp	0.34	**0.030**	0.40	**0.011**	0.31	**0.046**	0.57	**0.002**	0.01	0.460	-0.06	0.542
phenol oxidase	0.34	**0.034**	0.39	**0.013**	0.31	0.081	0.58	**0.004**	0.02	0.411	-0.06	0.520
cutinase	0.29	0.061	0.40	**0.005**	0.29	0.089	0.53	**0.003**	-0.02	0.505	-0.02	0.406
cdh	0.36	**0.008**	0.41	**0.013**	0.31	**0.042**	0.41	**0.013**	0.02	0.405	-0.05	0.517

[a]Bray-Curtis distances are used to measure dissimilarities.

[b]The targeted substrates are arranged in order from labile C to recalcitrant C.

[c]Significant differences with *p* values less than 0.050 are marked in bold.

Supplementary Table S5. Governing equations, component fluxes, and parameters in the MEND model

C pool variation	Governing equation	
Particulate organic matter (POM) decomposed by oxidative enzymes (*PO*)	$\frac{dPO}{dt} = I_{PO} + (1 - g_D) \cdot g_{PO} \cdot F_9 - F_1$ $I_{PO} + I_{PH} + I_D = I_{gross} \cdot fINP$, I_{gross} is the gross litter input	(S1)
POM pool decomposed by hydrolytic enzymes (*PH*)	$\frac{dPH}{dt} = I_{PH} + (1 - g_D) \cdot g_{PO} \cdot F_9 - F_2$	(S2)
Mineral-associated organic C (MOM, *M*)	$\frac{dM}{dt} = (1 - f_D) \cdot (F_1 + F_2) - F_3$	(S3)
Adsorbed DOM (QOM, *Q*)	$\frac{dQ}{dt} = F_4 - F_5$	(S4)
Dissolved organic C (DOM, *D*)	$\frac{dD}{dt} = I_D + f_D \cdot (F_1 + F_2) + g_D \cdot F_9 + F_{16} - F_6 - (F_4 - F_5)$	(S5)
MBA	$\frac{dBA}{dt} = F_6 - (F_7 - F_8) - F_9 - (F_{10} + F_{11} + F_{12}) - F_{15}$	(S6)
MBD	$\frac{dBD}{dt} = (F_7 - F_8) - (F_{13} + F_{14})$	(S7)
Enzymes for C pools	$\frac{dED_i}{dt} = F_{15,ED_i} - F_{16,ED_i}$, $ED_i (i = 1,2,3)$ denotes *EPO*, *EPH*, *EM*, respectively	(S8)
Respiration (R_h, R_a, R_S)	$R_h = (F_{10} + F_{11} + F_{12}) + (F_{13} + F_{14})$ $R_a = fR_a \times GPP$ $R_S = R_h + R_a$	(S9-11)
C balance	$\frac{d}{dt}\left(PO + PH + M + Q + D + BA + BD + \sum_{i=1}^{3} ED_i\right) = I_{PO} + I_{PH} + I_D - R_h$	(S12)
**Flux description**	**Equation**	

Particulate organic matter (POM) pool (oxidative, *PO*) decomposition (F_1)	$F_1 = Vd_{PO} \cdot EPO \cdot PO/(K_{PO} + PO)$	(S13)
POM pool (hydrolytic, *PH*) decomposition	$F_2 = Vd_{PH} \cdot EPH \cdot PH/(K_{PH} + PH)$	(S14)
Mineral-associated organic matter (MOM, *M*) decomposition	$F_3 = Vd_M \cdot EM \cdot M/(K_M + M)$	(S15)
Adsorption (F_4) and desorption (F_5) between dissolved organic matter (DOM, *D*) and adsorbed DOM (QOM, *Q*)	$F_4 = k_{ads} \cdot (1 - Q/Q_{max}) \cdot D$<br>$F_5 = k_{des} \cdot (Q/Q_{max})$<br>$k_{ads} = k_{des} \cdot k_{ba}$	(S16)<br>(S17)
DOM (*D*) uptake by microorganisms	$F_6 = \frac{1}{Y_g} \cdot (V_g + V_m) \frac{D \cdot BA}{K_D + D}$	(S18)
Dormancy (F_7) and reactivation (F_8) between active (BA) and dormant (BD) microorganisms	$F_7 = [1 - D/(K_D + D)] \cdot V_m \cdot BA$<br>$F_8 = D/(K_D + D) \cdot V_m \cdot BD$	(S19)<br>(S20)
MB_A (*BA*) mortality	$F_9 = \gamma \cdot V_m \cdot BA$	(S21)
MB_A (*BA*) growth respiration (F_{10}) and maintenance respiration (F_{11})	$F_{10} = (\frac{1}{Y_g} - 1) \frac{V_g \cdot D \cdot BA}{K_D + D}$<br>$F_{11} = (\frac{1}{Y_g} - 1) \frac{V_m \cdot D \cdot BA}{K_D + D}$	(S22)<br>(S23)
MB_A (*BA*) overflow respiration (F_{12})	$F_{12} = max\{0, BA - BAN \cdot CN_{BA,max}\}$	(S24)
MB_D (*BD*) maintenance respiration (F_{13})	$F_{13} = \beta \cdot V_m \cdot BD$	(S25)
MB_D (*BD*) overflow respiration (F_{14})	$F_{14} = max\{0, BD - BDN \cdot CN_{BD,max}\}$	(S26)

Synthesis of enzymes for decomposition of *PO* ($F_{15,\mathrm{EPO}}$, $EPO = ED_1$), *PH* ($F_{15,\mathrm{EPH}}$, $EPH = ED_2$), and *M* ($F_{15,\mathrm{EM}}$, $EM = ED_3$)	$F_{15,EPO} = PO/(PO+PH)\cdot p_{EP}\cdot V_m\cdot BA$<br>$F_{15,EPH} = PH/(PO+PH)\cdot p_{EP}\cdot V_m\cdot BA$<br>$F_{15,EM} = fp_{EM}\cdot p_{EM}\cdot V_m\cdot BA$<br>$F_{15} = \sum_{i=1}^{3} F_{15,ED_i}$	(S27)
Turnover of enzymes ($EPO = ED_1$, $EPH = ED_2$, $EM = ED_3$)	$F_{16,ED_i} = r_E\cdot ED_i$<br>$F_{16} = \sum_{i=1}^{3} F_{16,ED_i}$	(S28)

**Parameter**	**Description and units**[a]	**Parameter range**
LF_0	Initial fraction of *PO*, *LF0* = *PO*/(*PO*+*PH*)	(0.1, 1.0)
r_0	Initial active fraction of microorganisms	(0.01, 1)
fR_a	Scaling factor for autotrophic respiration (R_a)	(0.1, 0.4)
fINP	Scaling factor for litter input rate	(0.1, 0.9)
Vd	Maximum specific decomposition rate $Vd_{PO} = Vd_{PH} = Vd_M = Vd$. V_{PO}: Max specific decomposition rate for P_O; V_{PH}: Max specific decomposition rate for P_H; Vd_M: Max specific decomposition rate for *M*, mg C·mg^{-1} C·h^{-1}	(0.1, 100)
K_{PO}	Half-saturation constant (HSC) for *PO* decomposition, mg C·cm^{-3} soil	(40, 100)
fK_M	$K_M = K_{PO}\times fK_M$, $K_{PH} = K_{PO}/f_{KM}$, K_{PH} and K_M are HSC for P_H and *M*, respectively	(2, 20)
Q_{max}	Max sorption capacity, mg C·cm^{-3} soil	(0.5, 5)
K_{ba}	Binding affinity for DOM, (mg C·cm^{-3} soil)$^{-1}$	(1, 16)
k_{des}	Desorption rate for DOM, mg C·cm^{-3} soil·h^{-1}	(0.0001, 0.01)
r_E	Enzyme turnover rate, mg C·mg^{-1} C·h^{-1}	(0.0001, 0.01)
p_{EP}	[$V_m\times p_{EP}$] is the production rate of *EP* (EP_1+EP_2), V_m is the specific maintenance rate for active microorganisms	(0.0001, 0.05)
fp_{EM}	$fp_{EM}=p_{EM}/p_{EP}$, [$V_{mt}\times p_{EM}$] is the production rate of *EM*	(0.5, 3.0)
f_D	Fraction of decomposed *PO* and *PH* allocated to *D*	(0.05, 1)

g_D	Fraction of dead microorganisms allocated to D	(0.01, 1)
V_g	Max specific growth rate, mg C·mg^{-1} C·h^{-1}	(0.001, 0.1)
α	$= V_{mt}/(Vg + V_{mt})$, V_m is max specific maintenance rate	(0.01, 0.5)
K_D	HSC for microbial uptake of D, mg C·cm^{-3} soil	(0.01, 0.5)
Y_g	Intrinsic C use efficiency at reference temperature (T_{ref})	(0.1, 0.6)
k_{Yg}	Slope for Y_g dependence on temperature, (°C)$^{-1}$	(0.001,0.016)
γ	Max microbial mortality rate = $V_m \times \gamma$	(0.1, 20)
β	Ratio of dormant maintenance rate to V_m	(0.0005,0.05)
ψ_{A2D}	Soil water potential (SWP) threshold for microbial dormancy, MPa	(−0.6, −0.2)
τ	$\psi_{D2A} = \psi_{A2D} \times \tau$, ψ_{D2A} is the SWP threshold for microbial resuscitation	(0.1, 0.9)
ω	Exponential in SWP function for microbial dormancy or resuscitation	(1, 6)

[a]If not specified, the parameter is dimensionless

Supplementary Table S6. Objective functions used for different response variables in the MEND model parameterization.

Response variables	Description	Objective function
R_h	Heterotrophic respiration	Coefficient of determination (R^2) between simulated R_h and observed R_h
EnzCo	Concentration (EnzC) of oxidative enzyme	Correlation (*r*) between log-transformed simulated EnzC and observed relative gene abundance
EnzCh	Concentration (EnzC) of hydrolytic enzyme	Correlation (*r*) between log-transformed simulated EnzC and observed relative gene abundance

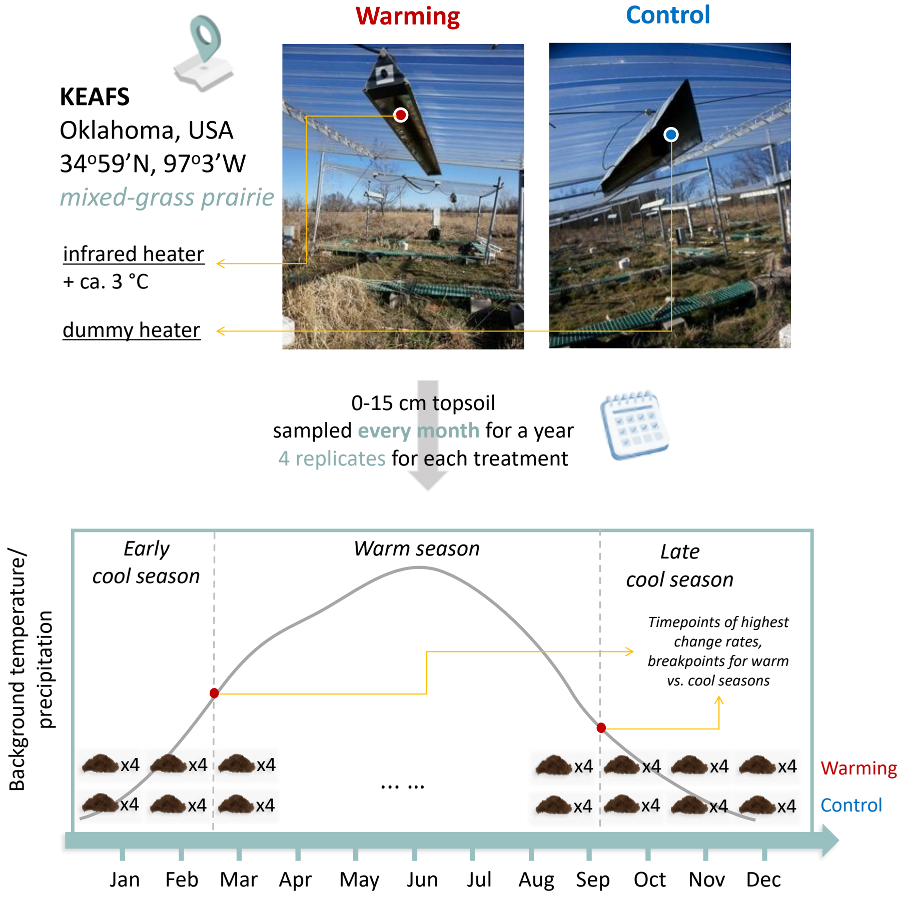

Supplementary Figure S1. A schematic map of the field experimental treatments and sampling scheme. The global warming experiment was initiated in July 2009, comprising four biological replicate blocks, each divided into two plots – one designated as the ambient control and the other as the warming (+3.0 °C) treatment as a paired design. Throughout the year 2012, which marked the 3rd year of experimental manipulation, monthly topsoils at a depth of 0–15 cm samples were collected in each of the eight plots. The year-round data were categorized into three seasons based on local temperature and precipitation patterns, enabling us to investigate the interactive effects of seasonality and warming.

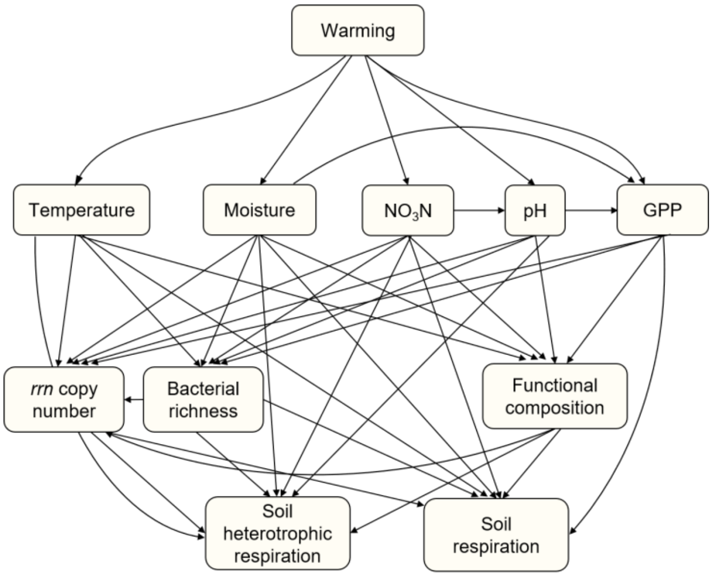

Supplementary Figure S2. *A priori* structural equation modeling (SEM) metamodel aimed to evaluate the link between warming and ecosystem functions. We assume that warming influences ecosystem functions (R_S and R_h) through alterations in environmental variables (soil temperature, soil moisture, NO_3^- content, and soil pH), plant communities (GPP), and microbial communities (bacterial richness, community-level *rrn* copy number, and functional composition represented by the first-axis scores from NMDS). The fitted models for the cool and warm season were available in Fig. 5.

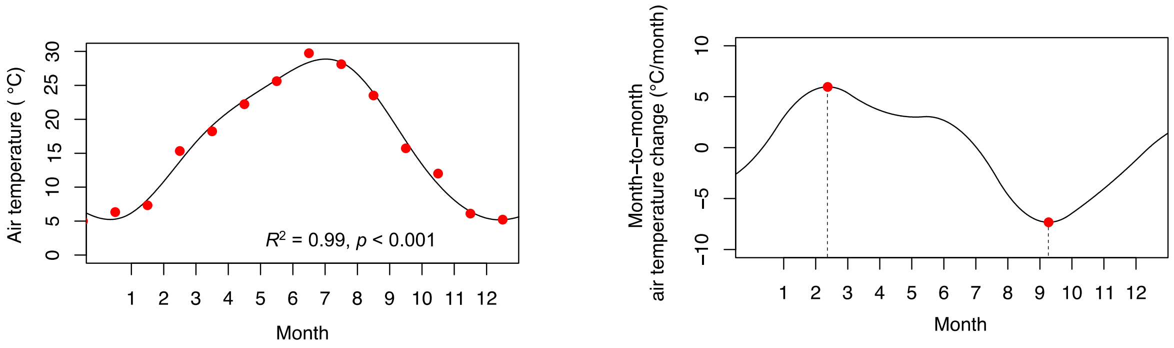

Supplementary Figure S3. Local temperature variability. **(A)** Temporal dynamics of monthly air temperature at KAEFS new warming site for 2012. Spline regression was used to approximate the temporal trend of air temperature, as denoted by the black line. Red dots denote monthly temperature measurements. **(B)** Temporal dynamics of air temperature change. The air temperature change rate was calculated as the first derivative of the temporal trend of air temperature. Red dots denote the fastest rise and decrease in temperature within the year.

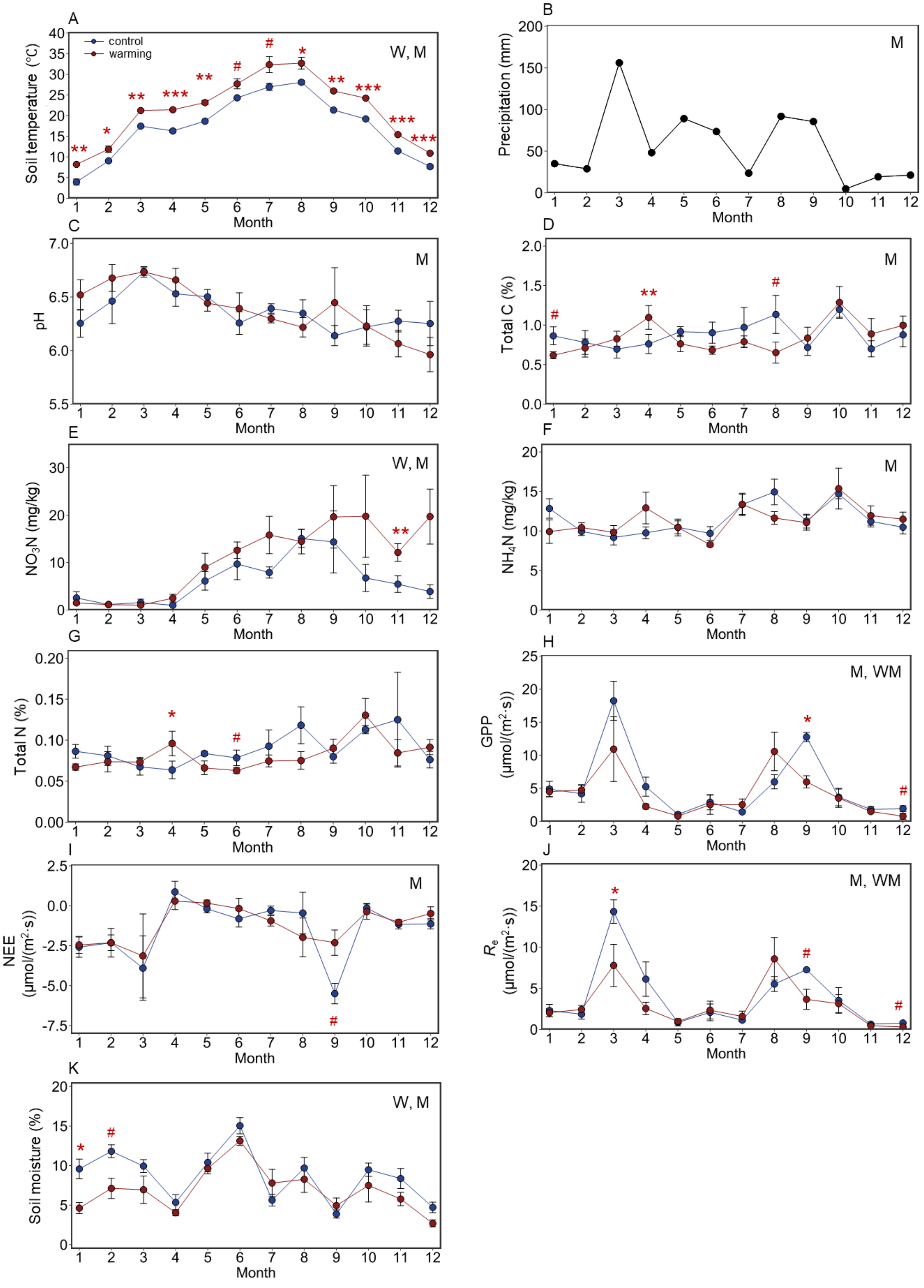
A
control
warming
W, M
Soil temperature (°C)
Month
B
M
Precipitation (mm)
Month
C
M
pH
Month
D
M
Total C (%)
Month
E
W, M
NO3N (mg/kg)
Month
F
M
NH4N (mg/kg)
Month
G
Total N (%)
Month
H
M, WM
GPP
(μmol/(m2·s))
Month
I
M
NEE
(μmol/(m2·s))
Month
J
M, WM
Re
(μmol/(m2·s))
Month
K
W, M
Soil moisture (%)
Month

Supplementary Figure S4. Monthly changes of environmental variables, ecosystem C fluxes, and R_S under warming and control. Dots represent the averaged values for measurements in each month from 4 replicated plots in (**A**-**K**). Soil temperature was measured at a depth of 7.5 cm. Precipitation was recorded on a monthly basis, with identical mean and s.e. for control and warming groups. R_e denotes ecosystem respiration. Negative values of gross primary production (GPP) and net ecosystem exchange (NEE) indicate C input for soil and vice versa. Differences between monthly means of control and warming were examined by paired *t*-tests (n = 4). Significances are indicated by *** when $p < 0.001$, ** when $p < 0.010$, * when $p < 0.050$, and # when $p < 0.100$. Analysis of variance (ANOVA) was employed to assess the effects of warming (W), months (M), and their interaction (WM). Those letters are indicated in the upper right corner of each panel if they have statistically significant effects ($p < 0.050$).

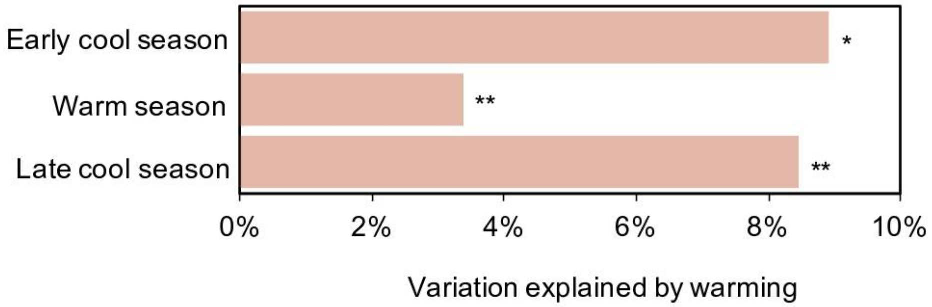

Supplementary Figure S5. Warming effect on microbial taxonomic composition across seasons. The percentage of variation in microbial taxonomic compositions explained by warming treatment in the early cool season, warm season, and late cool season, as tested by Adonis. Significances are indicated by ** when $p < 0.010$ and * when $p < 0.050$.

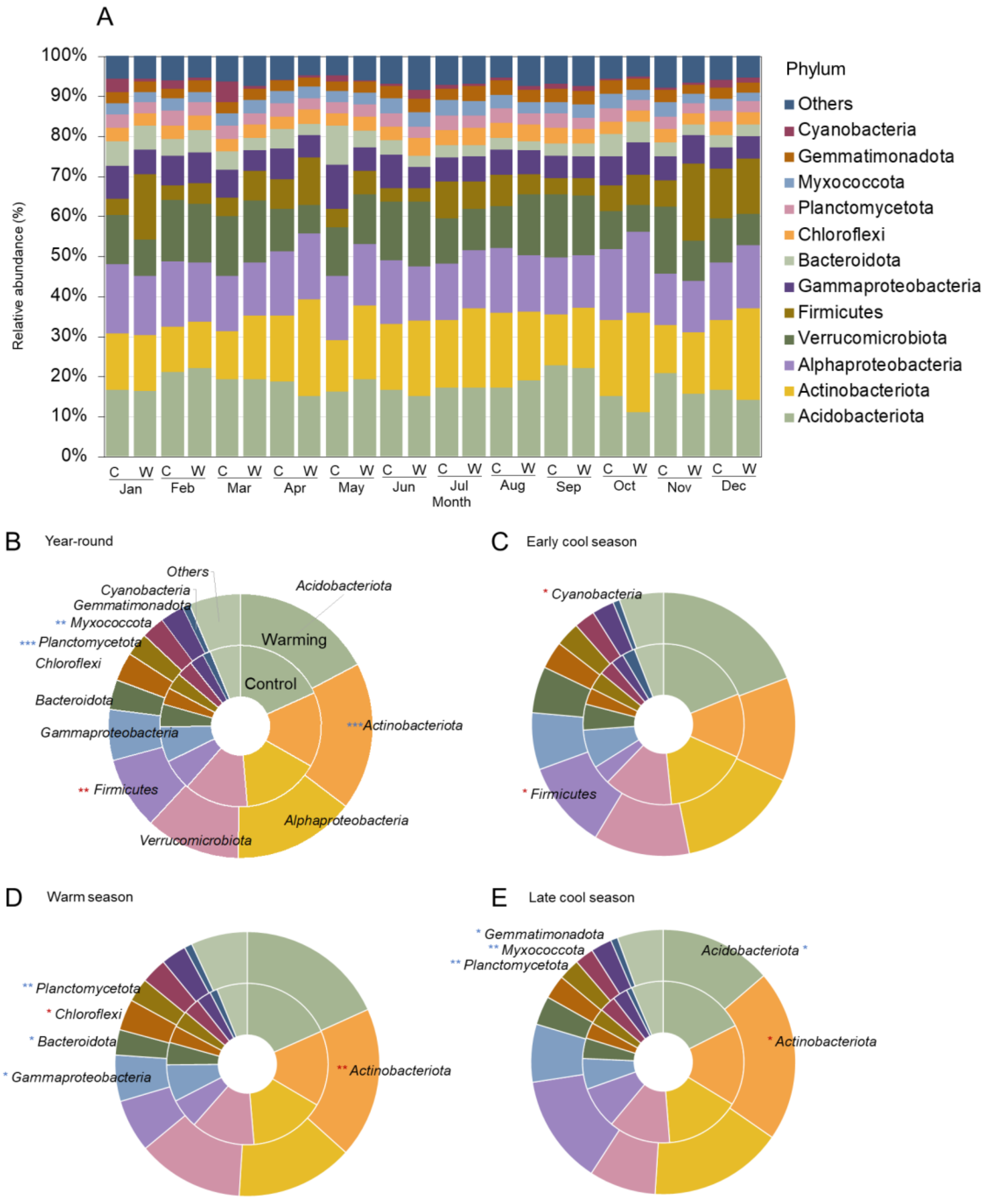

Supplementary Figure S6. Temporal dynamics of microbial taxonomic compositions at phylum level under warming and control. (A) Monthly dynamics of relative abundances of phyla (classes for *Proteobacteria*) in control (C) or warmed (W) plots. **(B-E)** Changes in relative abundances of phyla under warming (outer cycle) and control (inner cycle) treatments across all

seasons or in each season. Red asterisks indicate phyla with a significant increase of relative abundance by warming, while blue asterisks indicate phyla with a significant decrease of relative abundance by warming. The differences between warming and control were examined by linear mixed models with ANOVA and are shown by *** when $p < 0.001$, ** when $p < 0.010$, and * when $p < 0.050$.

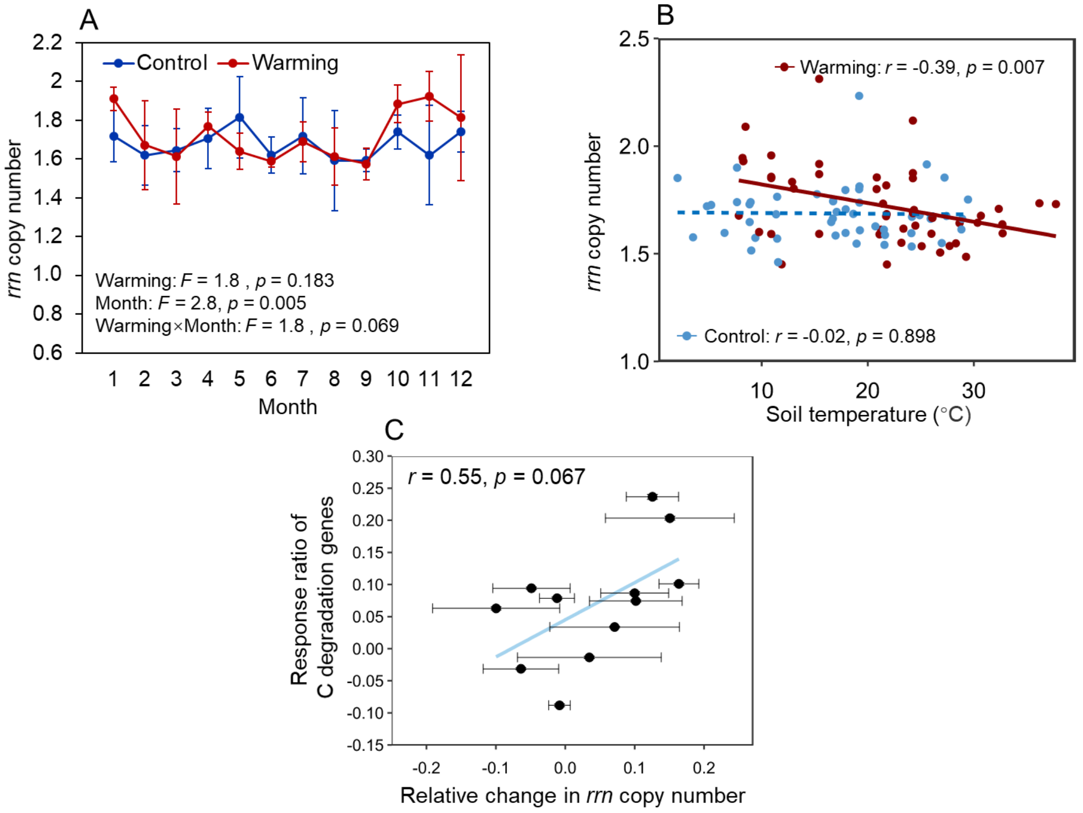

Supplementary Figure S7. Temporal dynamics of community average *rrn* copy number. (A) Community average *rrn* copy number, calculated as the sum of the abundance weighted *rrn* copy number of each ASV in a sample, on a monthly basis. Data are presented as means ± standard error (n = 4). The statistical significance of warming, month, and their interactions were tested by linear models with ANOVA. The *F* and *p* values are shown. **(B)** Correlations between monthly soil temperature and community average *rrn* copy number in control and warmed plots. Pearson correlation coefficient (r) and *p* value are shown. **(C)** Correlation between relative changes in community average *rrn* copy number by warming and response ratios showing warming effects on C-decomposing gene abundances. Relative changes in community average *rrn* copy number by warming are calculated as the logarithmic differences between average *rrn* copy number in warmed plots and control samples, and then averaged across 4 blocks for each month. Bidirectional

error bars denote standard errors (n = 4). Spearman correlation coefficient (*r*) and *p* value were calculated for n = 12 months.

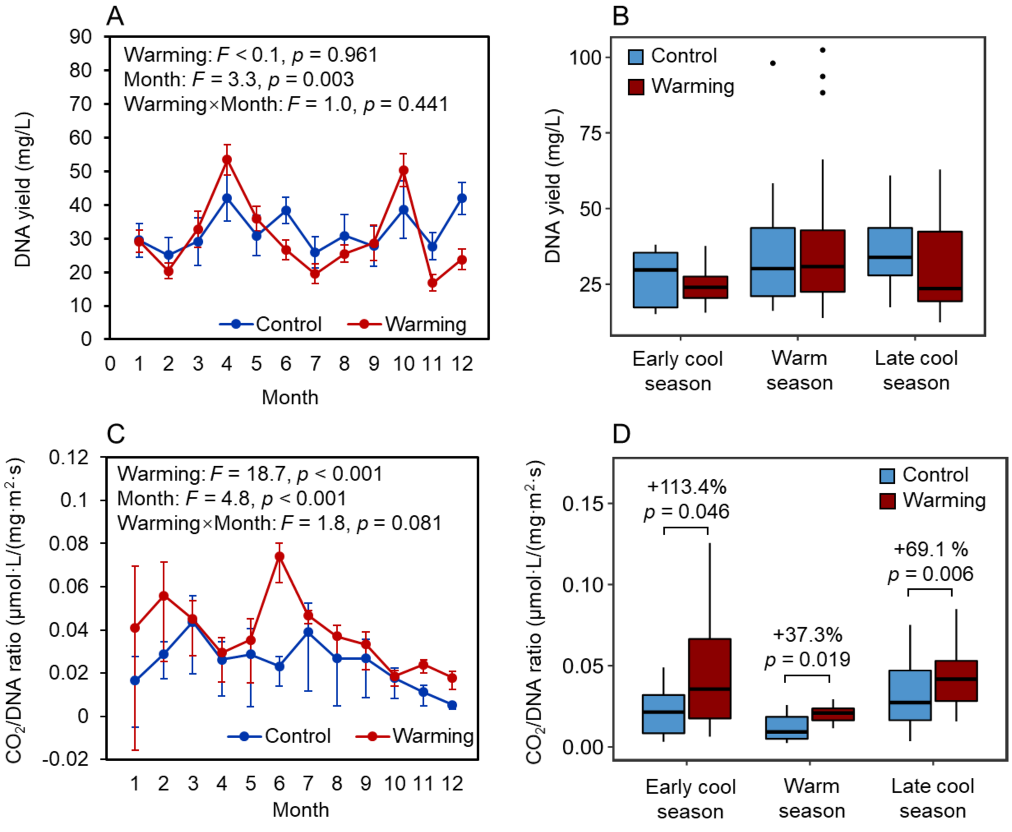

Supplementary Figure S8. (A) Monthly temporal variations of soil DNA yield. **(B)** DNA yield under control and warming in the early cool season (Jan to Feb), warm season (Mar to Sep), and late cool season (Oct to Dec). The differences between control and warming treatment were tested with paired *t*-tests. Only significant differences are marked. **(C)** The monthly microbial metabolic quotient at the community level, calculated as R_h/DNA ratios. **(D)** Microbial metabolic quotient in the early cool season (Jan to Feb), warm season (Mar to Sep), and late cool season (Oct to Dec). For (A,C), data are presented as means ± standard error (n = 4). The statistical significance of warming, month, and their interactions were tested by linear models with ANOVA. The F and *p* values are shown. For (B,D), The differences between control and warming treatment were tested with paired *t*-tests. Only significant differences are shown.

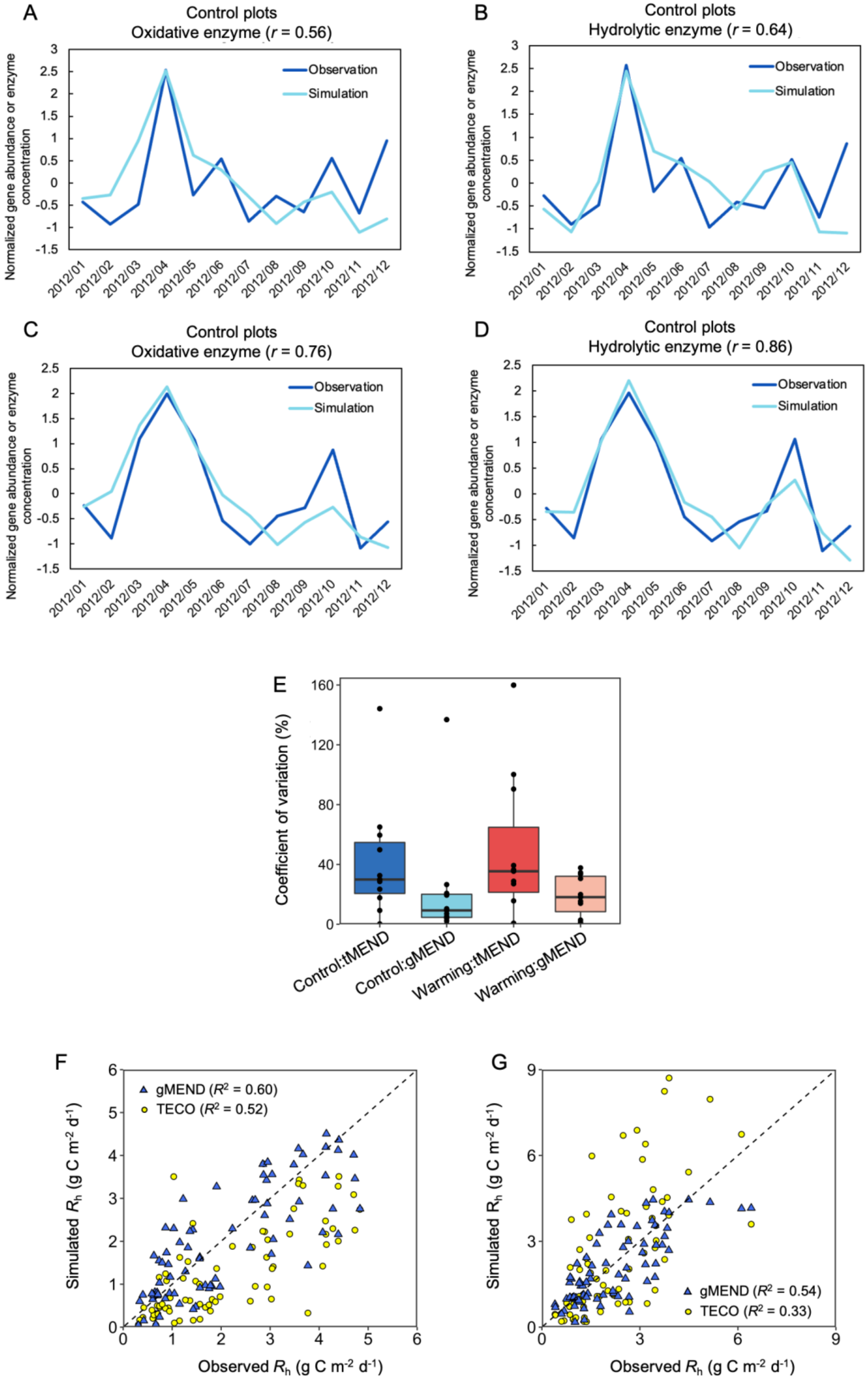
A
Control plots
Oxidative enzyme (r = 0.56)
Observation
Simulation
Normalized gene abundance or enzyme concentration
B
Control plots
Hydrolytic enzyme (r = 0.64)
C
Control plots
Oxidative enzyme (r = 0.76)
D
Control plots
Hydrolytic enzyme (r = 0.86)
2012/01
2012/02
2012/03
2012/04
2012/05
2012/06
2012/07
2012/08
2012/09
2012/10
2012/11
2012/12
E
Coefficient of variation (%)
Control:tMEND
Control:gMEND
Warming:tMEND
Warming:gMEND
F
gMEND (R2 = 0.60)
TECO (R2 = 0.52)
Simulated Rh (g C m-2 d-1)
Observed Rh (g C m-2 d-1)
G
gMEND (R2 = 0.54)
TECO (R2 = 0.33)

Supplementary Figure S9. Evaluation of MEND modeling performance with gene abundance data. (A,B) gMEND-simulated enzyme concentrations vs. GeoChip-detected gene abundances for (A) oxidative enzymes and (B) hydrolytic enzymes in the control plot. **(C,D)** gMEND-simulated enzyme concentrations vs. GeoChip-detected gene abundances for (A) oxidative enzymes and (B) hydrolytic enzymes in the warmed plot. Model performance for the control plot is quantified by the correlation coefficient (*r*), as the absolute values between GeoChip gene abundances and MEND enzyme concentrations cannot be directly compared. **(E)** The MEND model parameter uncertainty quantified by the Coefficient of Variation (CV). The bars show the mean CV values of the 11 parameters (See Supplementary Table S3 for details). The dots along each bar show the CV for each parameter. tMEND refers to the traditional MEND model parameterization without gene abundance data. gMEND denotes the improved MEND parameterization with gene abundances. **(F, G)** Comparison of model performance between gMEND and the non-microbial model TECO in (F) control plots and (G) warmed plots.
